# Aging Potentiates Lateral but Not Local Inhibition of Orientation Processing in Primary Visual Cortex

**DOI:** 10.3389/fnagi.2018.00014

**Published:** 2018-02-05

**Authors:** Zhengchun Wang, Shan Yu, Yu Fu, Tzvetomir Tzvetanov, Yifeng Zhou

**Affiliations:** ^1^Hefei National Laboratory for Physical Sciences at Microscale and School of Life Science, University of Science and Technology of China, Hefei, China; ^2^Department of Physiology and Pharmacology, Medical School of Ningbo University, Ningbo, China; ^3^Brainnetome Center and National Laboratory of Pattern Recognition, Institute of Automation, and Center for Excellence in Brain Science and Intelligence Technology, Chinese Academy of Sciences, Beijing, China; ^4^Department of Basic Medicine, Medical School, Kunming University of Science and Technology, Kunming, China; ^5^School of Computer and Information, Hefei University of Technology, Hefei, China; ^6^State Key Laboratory of Brain and Cognitive Science, Institute of Biophysics, Chinese Academy of Sciences, Beijing, China

**Keywords:** behavioral measure, computational modeling, neurophysiology, early visual processing, senescence

## Abstract

Aging-related declines in vision can decrease well-being of the elderly. Concerning early sensory changes as in the primary visual cortex, physiological and behavioral reports seem contradictory. Neurophysiological studies on orientation tuning properties suggested that neuronal changes might come from decreased cortical local inhibition. However, behavioral results either showed no clear deficits in orientation processing in older adults, or proposed stronger surround suppression. Through psychophysical experiments and computational modeling, we resolved these discrepancies by suggesting that lateral inhibition increased in older adults while neuronal orientation tuning widths, related to local inhibition, stayed globally intact across age. We confirmed this later result by re-analyzing published neurophysiological data, which showed no systematic tuning width changes, but instead displayed a higher neuronal noise with aging. These results suggest a stronger lateral inhibition and mixed effects on local inhibition during aging, revealing a more complex picture of age-related effects in the central visual system than people previously thought.

## Introduction

Aging has a profound impact on human visual function, where early neuronal processing and perceptual abilities modifications with age have been intensively studied in the last 25 years (Owsley, 2011; Andersen, 2012). Age-related changes in cortical inhibition have been suggested to be involved in visual functions decline. But as the field currently stands, there is no clear unifying account of early stages changes as in spatial vision.

Neurophysiological studies suggested that a reduction in inhibitory function might be related to age-related perceptual dysfunctions in spatial vision (Schmolesky et al., 2000; Leventhal et al., 2003). Nevertheless, some behavioral studies have shown that there are low-level processes of visual functions which are inconsistent with the physiological interpretation of decreased inhibition: (1) psychophysical sensitivities to orientation processing in older populations are not systematically and substantially changed when compared to younger adults (Betts et al., 2007; Delahunt et al., 2008; Govenlock et al., 2009), while the neurophysiological counterpart strongly suggests such a deficit; (2) contrast surround suppression in older adults was found increased, which was interpreted with stronger inhibitory interactions (Karas and McKendrick, 2015). Surround suppression could be corresponding to neurons' response with stimuli at extra classical receptive field (extra-CRF), involving lateral inhibitory interactions between cortical “channels” (Gilbert, 1992; Spillmann and Werner, 1996; Angelucci et al., 2002; Angelucci and Bressloff, 2006), while neurophysiological inference was drawn from classical receptive field (CRF) properties of cells in the visual cortex (area V1). Since the changed cellular properties are commonly attributed to intracortical inhibition (Somers et al., 1995; Shapley et al., 2003), it appears that intracortical local inhibition and lateral inhibition could have underwent heterogeneous modifications with old age.

**Figure 1:**
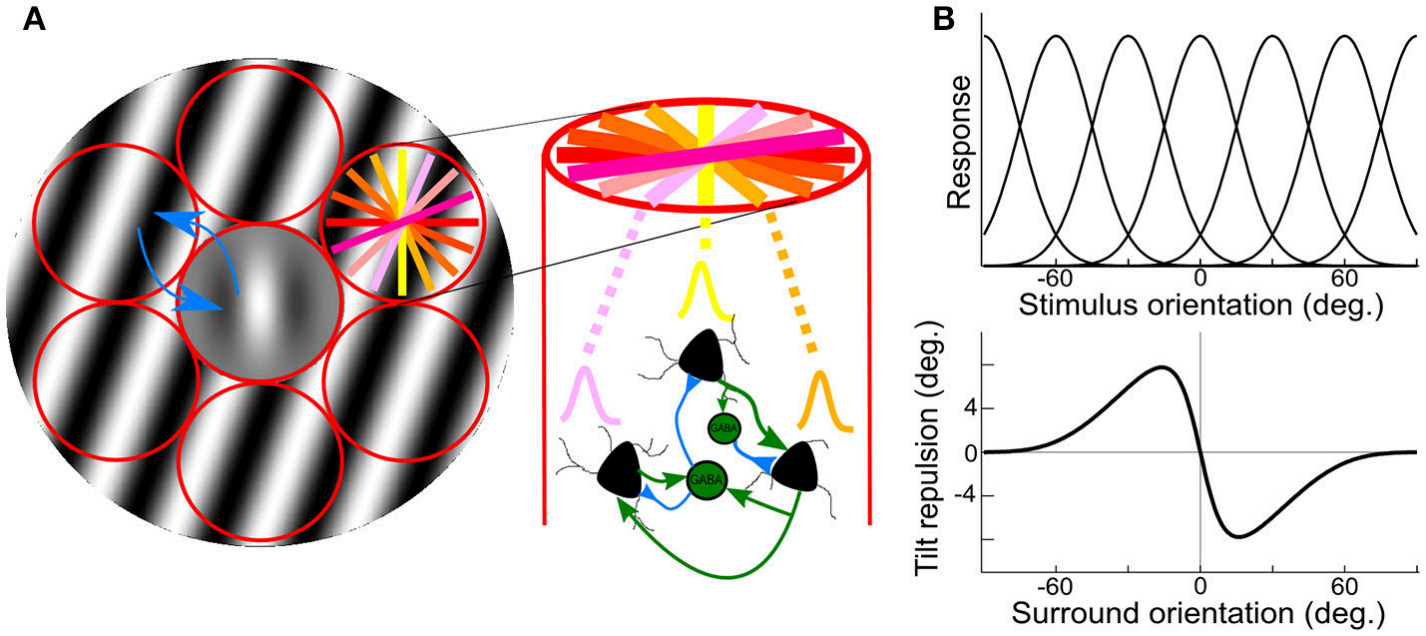
Illustration of center-surround stimulus, orientation hypercolumns, population tuning curves and behavioral outcome. **(A)** Illustration of the stimulus, a center Gabor patch surrounded by an annulus of oriented grating, padded with orientation hypercolumns (red circles); the colored lines in right-top circle depict different preferred orientations of the local neuronal population; on the right, illustration of local interaction within an orientation hypercolumn, with three example neurons with different preferred orientations. Two inter-neurons, and their local connections (blue/green are inhibitory/excitatory connections); blue arrows depict inhibitory lateral interactions between hypercolumns. **(B)** Theoretical population orientation tuning curves (top) together with typical tilt repulsion curve (bottom) describing the orientation misperception of a vertical center stimulus as a function of the orientation of the surround; zero is vertical and positive values are clockwise tilts.

These two levels of interactions can be accessed non-invasively through the use of psychophysics measures and computational modeling (Gilbert and Wiesel, 1990; Tzvetanov and Womelsdorf, 2008; Tzvetanov, 2012). We used the tilt illusion (TI) paradigm where the presence of an orientated surround stimulus biases the perceived orientation of a simultaneously presented center test. Current opinions propose that tilt repulsion effects can be explained through lateral inhibition between spatially arranged orientation hypercolumns of neurons in V1 (Georgeson, 1973; Wenderoth and Smith, 1999; Kapadia et al., 2000; Figure 1A, red). Specifically, in the classical center-surround tilt repulsion effect (Gilbert and Wiesel, 1990; Angelucci and Bressloff, 2006; Figure 1A), one can infer the local interactions through the indirect measure of the orientation tuning widths of a theoretical population of neurons (Figures 1A,B, top), which is directly related to the local inhibition within one hypercolumn (Somers et al., 1995), but also the lateral interactions strengths between different hypercolumns (blue arrows in Figure 1A). Both local and lateral interactions influence the tilt repulsion curve (Figure 1B, bottom), that describes the misperception of the subject of a vertically oriented center target when the orientation of the surround is varied (Figure 1A, gray stimulus). Through model adjustment to the behavioral data, estimated mean values of these physiological variables of the neural population was accessed for each person.

Here, we carried out psychophysical measures in younger and older populations in order to characterize their center-surround tilt repulsion at two spatial frequencies (SFs), one near best perceptual sensitivity and one at higher SF, obtained from the individual contrast sensitivity function (CSF). Then, we performed modeling of subjects' perception assuming it is obtained from decoding these V1 neuronal activities. Contrary to the results from old adult animals suggesting that age-related changes of visual perception in older adults might stem from a reduction in inhibitory functions, the combination of psychophysics measures and modeling showed different aging-related effects on different kinds of inhibition: (1) an increased lateral inhibition with age and (2) maintained local orientation tuning widths, and thus local inhibition (Somers et al., 1995; Carandini and Ringach, 1997; Shapley et al., 2003), of the neuronal population in older adults. To investigate further this later result, we re-analyzed previously published physiological data about orientation processing from our laboratory and extracted the tuning width of neurons. These physiological results are consistent with the behavioral and computational findings of unchanged orientation tuning widths.

## Results

### Contrast sensitivity functions

**Figure 2:**
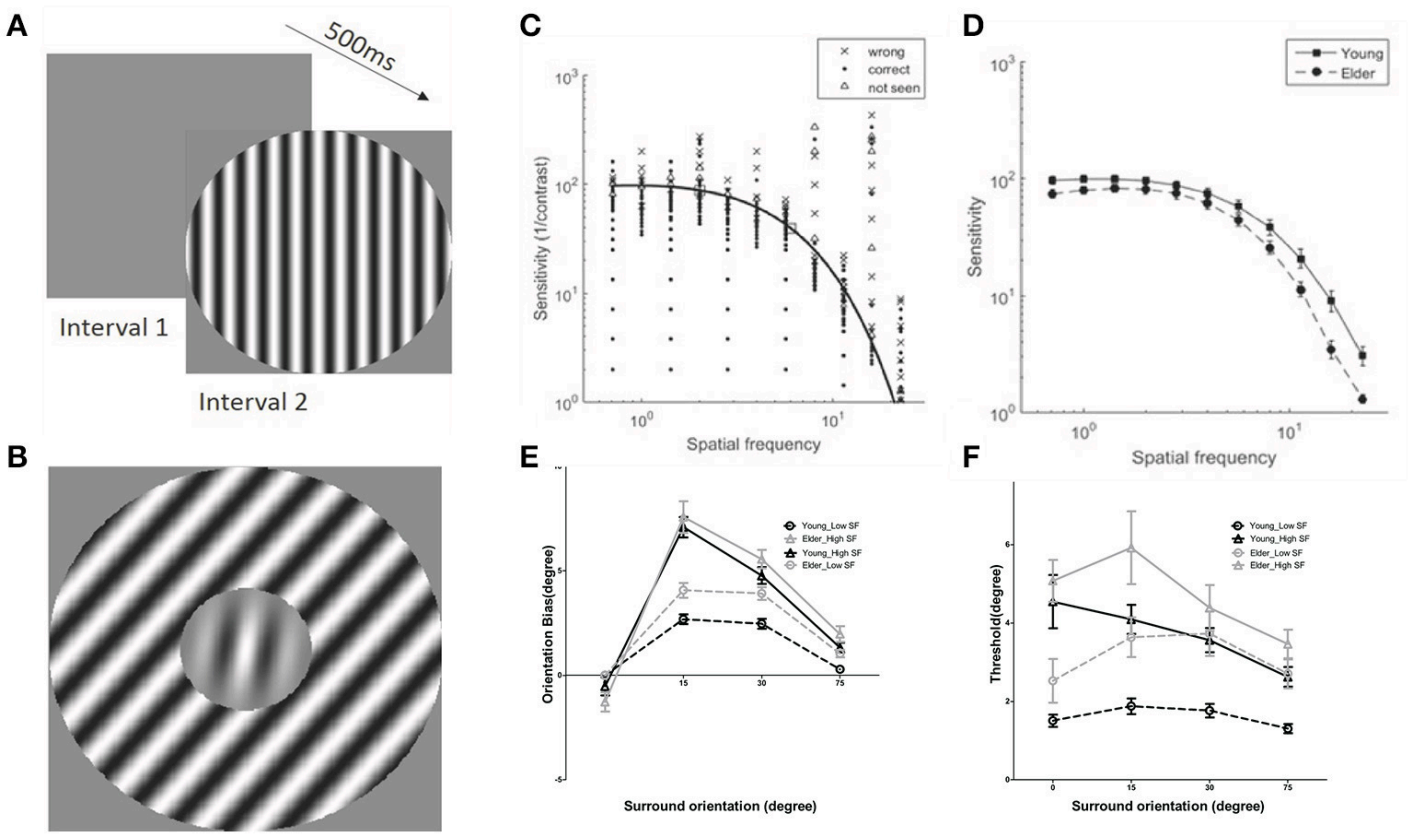
Examples of stimuli used and results of contrast sensitivity function and tilt illusion measures. **(A)** Schematic illustration of the contrast sensitivity function (CSF) measure; **(B)** Example of stimuli in tilt illusion measure. **(C)** Examples of results of CSF measures and fits in the 2-Alternative unforced Choice (2AuFC) design; squares depict the two chosen SFs for the subsequent tilt measures; **(D)** Averaged values of contrast sensitivities at the 11 different SFs measured for older adults and younger groups; **(E)** Tilt illusion results, indicated by orientation bias necessary to perceive the center as vertical, as a function of SOs and SFs (low-SF: circles; high-SF: triangles) for older adults (gray) and younger (black) groups; negative (positive) deviations for negative (positive) surround orientations correspond to repulsion; **(F)** Orientation thresholds around perceived verticality.

Contrast sensitivity function (CSF), representing the inverse of the minimum detectable contrast at each spatial frequency (SF), was measured for each subject prior to the TI measure (Figures 2A,C,D). The contrast sensitivity (CS) was significantly modulated by SF [*F*_(10, 380)_ = 599.56, *P* < 0.0001] and CS of the elderly group was significantly lower than the young adults group across all spatial frequencies [*F*_(1, 38)_ = 7.44, *P* < 0.05], confirming previous reports(Owsley, 2011). The interaction of the SF and groups was also significant [*F*_(10, 380)_ = 7.23, *P* < 0.01]. From each individual CSF, we chose two spatial frequencies (SFs), one near the peak sensitivity (low-SF) and one higher (high-SF) (Figure 2C, squares), with the condition that the sensitivity at the high-SF was not too low for allowing stimulus perception at next stage of measures of center-surround TI.

### Tilt illusion measurement

Perceived verticality of the target grating under center-surround orientation differences of 0, ±15, ±30, and ±75° for each subject was measured at low- and high-SF (Figure 2B). The amount of orientation misperception for both groups was systematically modulated by surround orientations (SOs) and SFs [Surr. Or.: *F*_(3, 114)_ = 240.41, *P* < 0.001; SF: *F*_(1, 38)_ = 75.14, *P* < 0.001] (Figure 2E), consistent with previous reports (Georgeson, 1973; Wenderoth and Johnstone, 1988; Smith and Wenderoth, 1999). Importantly, analysis limited to ±15° and ±30° SOs, corresponding to the direct form of the TI (Wenderoth and Johnstone, 1988; Smith and Wenderoth, 1999; Wenderoth and Smith, 1999), repulsion, revealed that perception through older adults' visual system exhibited much stronger tilt repulsion effects across all measured SFs in comparison to the younger adults [*F*_(1, 38)_ = 6.62, *P* < 0.05; Figure 2E]. The amount of misperception at ±15° SO was significantly different than that at ±30° [*F*_(1, 38)_ = 53.79, *P* < 0.0001]. There were no significant interactions between SOs and groups [*F*_(1, 38)_ = 0.32, *P* = 0.57] indicating similar young-elder differences at both SOs. The amount of misperception for low- and high-SFs across groups was significantly modulated [*F*_(1, 38)_ = 89.72, *P* < 0.001], but there was no interaction between SFs and groups [*F*_(1, 38)_ = 1.56, *P* = 0.22] that suggested same effects of SFs across age groups. There was a significant interaction between SOs and SFs [*F*_(1, 38)_ = 41.28, *P* < 0.001], while there were no significant interactions among SOs, SFs and Groups [*F*_(1, 38)_ = 0.15, *P* = 0.70], again showing no differences of effects across age.

Orientation discrimination thresholds in elder group were larger than younger adults [*F*_(1, 38)_ = 7.25, *P* < 0.05], and they were modulated by SOs [*F*_(3, 114)_ = 11.86, *P* < 0.0001] and SFs [*F*_(1, 38)_ = 49.66, *P* < 0.0001]. There was also a main interaction between SOs and SFs [*F*_(3, 114)_ = 7.58, *P* < 0.001] showing a different trend of threshold variation with surround orientation at different SFs (Figure 2F). All other interactions that included age group were not significant [SOs × Groups: *F*_(3, 114)_ = 1.73, *P* = 0.17; SFs × Groups: *F*_(1, 38)_ = 1.04, *P* = 0.32; SOs × SFs × Groups: *F*_(3, 114)_ = 1.73, *P* = 0.17]. We would like to emphasize that the age effect on thresholds does not allow to draw a direct conclusion that there are different orientation threshold between younger and older groups, due to no adjustment of these threshold values to the individual sensitivity (see Discussion).

**Figure 3:**
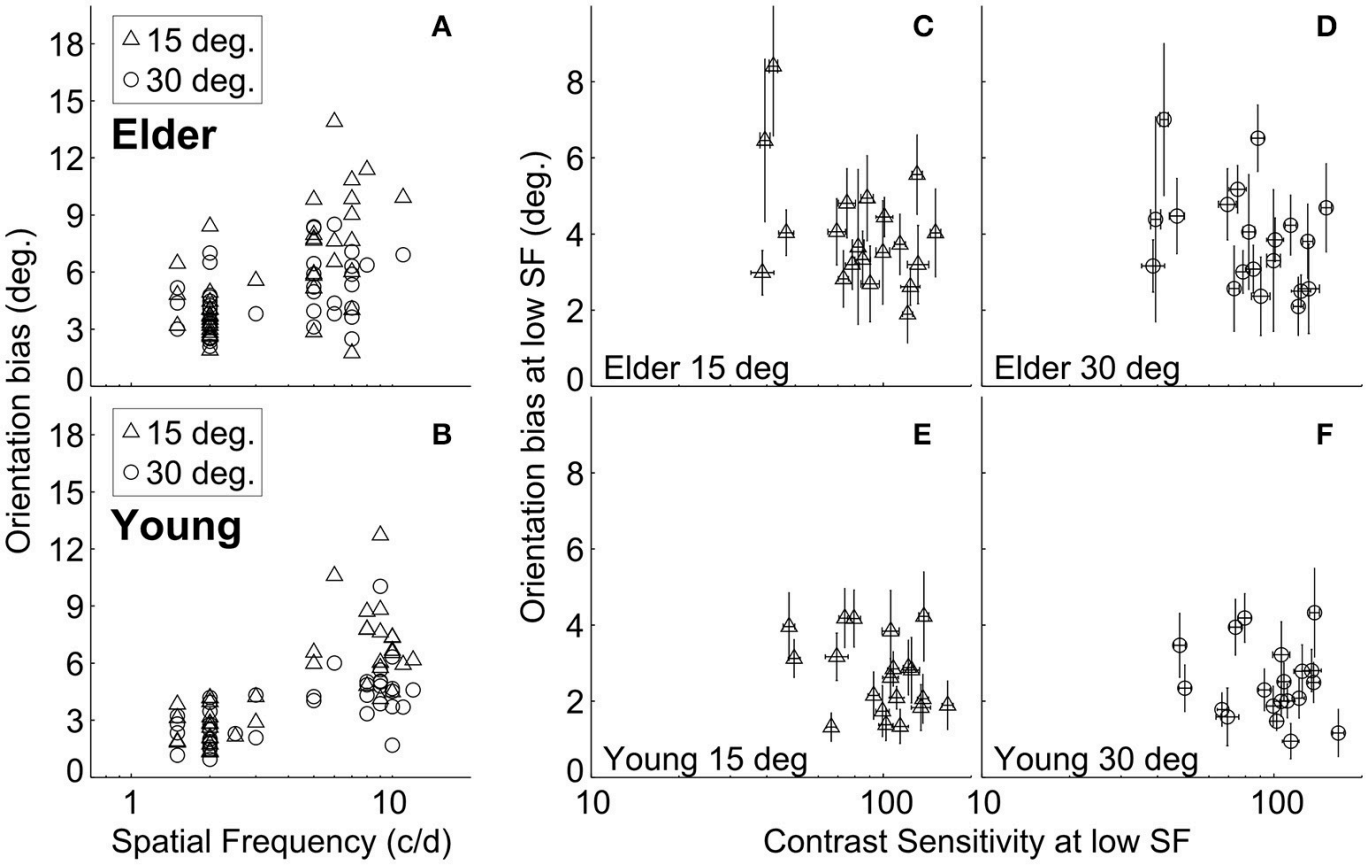
Relation between tilt repulsion, SF and CS at low-SF. Relations between tilt repulsion and SFs under ±15° (triangles) and ±30° (circles) SOs across all measured subjects in older adults **(A)** and younger **(B)** groups; Relations between tilt repulsion and CS at low-SF in the elderly group [±15° in **(C)**; ±30° in **(D)**] and younger (±15° in **(E)**; ±30° in **(F)**] groups. Error bars are bootstrapped SE.

It is possible that the different high-SF used in younger and elder groups contributed to the differences in misperception between them. We found, as in previous work (Smith and Wenderoth, 1999), that the amount of orientation bias was significantly related to the SF under both ±15° and ±30° SOs conditions for both elder and younger groups (Spearman rank's correlation; the elder: *r* = 0.58, *P* = 8.5^*^10^−5^ at ±15°, *r* = 0.39, *P* = 0.013 at ±30°; the younger adults: *r* = 0.74, *P* = 3.6^*^10^−8^ at ±15°, *r* = 0.63, *P* = 1.2^*^10^−5^ at ±30°) (Figures 3A,B). This suggested that adjusting for the variation of TI with SF (Smith and Wenderoth, 1999) still showed that older adults exhibited stronger misperception. Additionally, the old persons group had globally lower high-SF [*t*_(38)_ = 5.37, *P* < 0.0001, unpaired *t*-test] than the younger group, showing that their stronger repulsion effects cannot be explained by this experimental manipulation.

### Relation between tilt illusion bias and contrast sensitivity

The elderly group exhibited decreased contrast sensitivity and increased amount of TI compared to the younger adults. To reveal any possible relation between tilt repulsion and peak CS, we correlated tilt and CS measured at low-SF condition, where the SF values for both groups are similar. The results showed that there was no significant relation between bias and CS within each age group (the elder: *r* = −0.25, *P* = 0.29 at ±15°, *r* = −0.36, *P* = 0.12 at ±30°; the younger adults: *r* = −0.21, *P* = 0.38 at ±15°, *r* = −0.02, *P* = 0.92 at ±30°) (Figures 3C–F).

### Modeling

**Figure 4:**
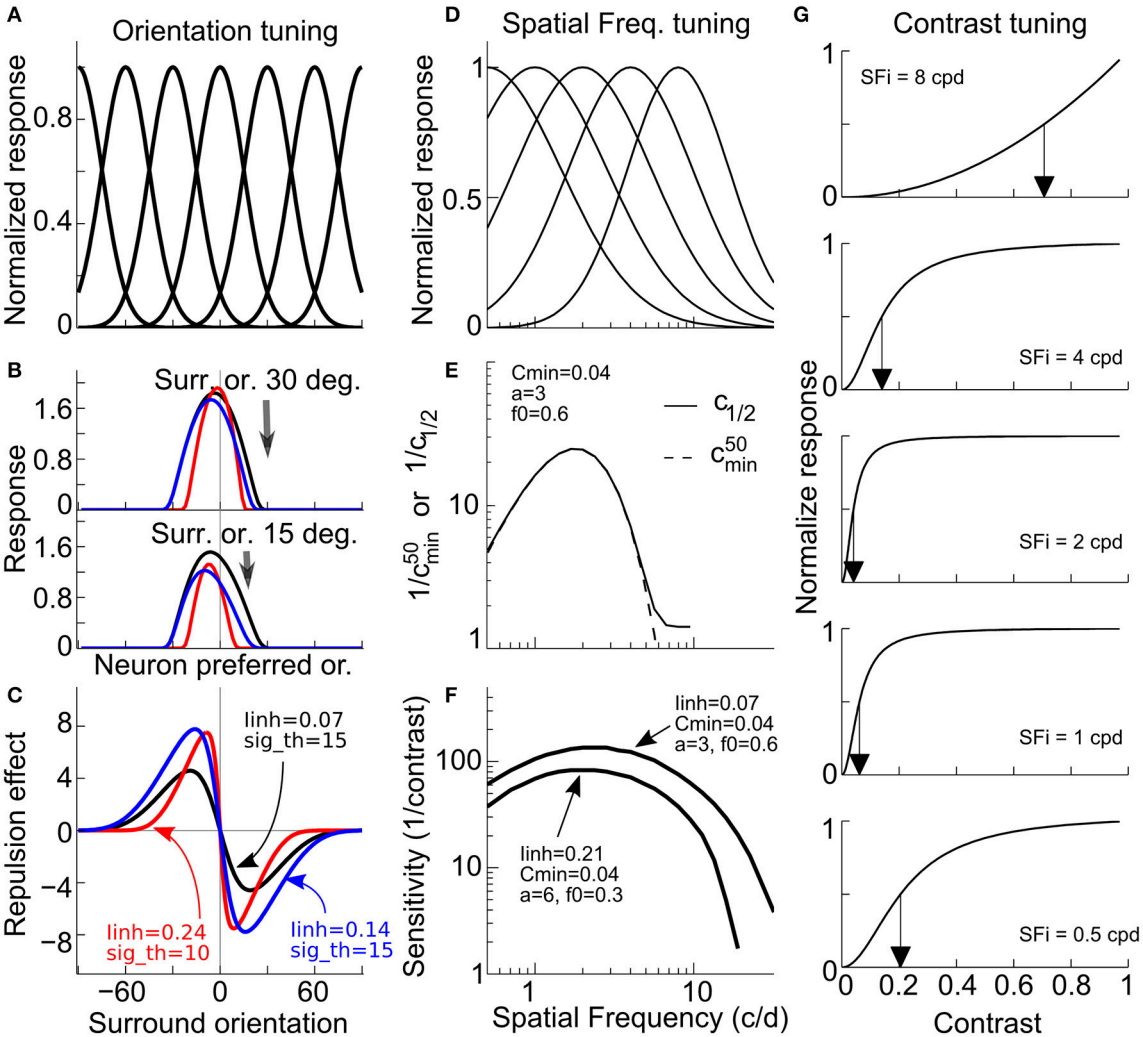
V1 model illustration. **(A–C)** Example and prediction for orientation coding and decoding. **(A)** Uniform orientation tuning of the neuronal population. **(B)** Response of the neuronal population to center of 0°. and two different surround orientations of ±30° (top) and ±15° (bottom). **(C)** Orientation prediction of the model from the population responses for various surround orientations. Examples for three different set of parameters in red, black and blue. **(D–G)** Example and prediction for (SF) and contrast tuning coding and decoding. **(D)** SF tuning examples, with the characteristic tuning width decrease with increasing preferred SF. **(E)** Example of the relation between the minimum contrast semi-saturation constant and preferred SF. **(F)** Examples of CSF prediction for two sets of model parameters. **(G)** Examples of contrast response functions in the model for the minimum semi-saturation constant at few preferred SFs (arrows depict half-amplitude constant).

To further our understanding of early visual processing and the plausible underlying network changes with aging, based on the learned knowledge that neuronal activity in V1 could be the substrate of orientation identification and detection (Georgeson, 1973; Gilbert and Wiesel, 1990; Kapadia et al., 2000; Tzvetanov, 2012), we modeled tilt repulsion effects and contrast sensitivity by including the spatial lateral inhibition in V1. Psychophysical modeling based on the V1 neuronal responses relates each behavioral variable to different tuning characteristics: orientation misperception is obtained through orientation tuning and center-surround inhibitory interactions (Figures 4A–C), while CSF could be obtained through the SF and contrast tuning of the neurons (Figures 4D–G).

To obtain an insight into the differences between older adults and younger adults (for instance, lateral inhibition, orientation tuning, contrast tuning or a combination of them), we modeled a two-layer feed-forward surround-to-center inhibitory network of V1 neurons. In this model, the TI and contrast sensitivity function (Figures 4C,F) are related through a single parameter, the amount of surround-to-center inhibition (defined as *I*_*inh*_). The amplitude and shape of the orientation misperception are dependent on *I*_*inh*_ and orientation tuning width (defined as σ_θ_) (Figures 4A–C). The CSF is dependent on *I*_*inh*_ (because the grating stimulus excites center and surround mechanisms) together with the smallest contrast semi-saturation constant (defined as *c*_*min*_) and its relation to the SF tuning (Figures 4D–G).

**Figure 5:**
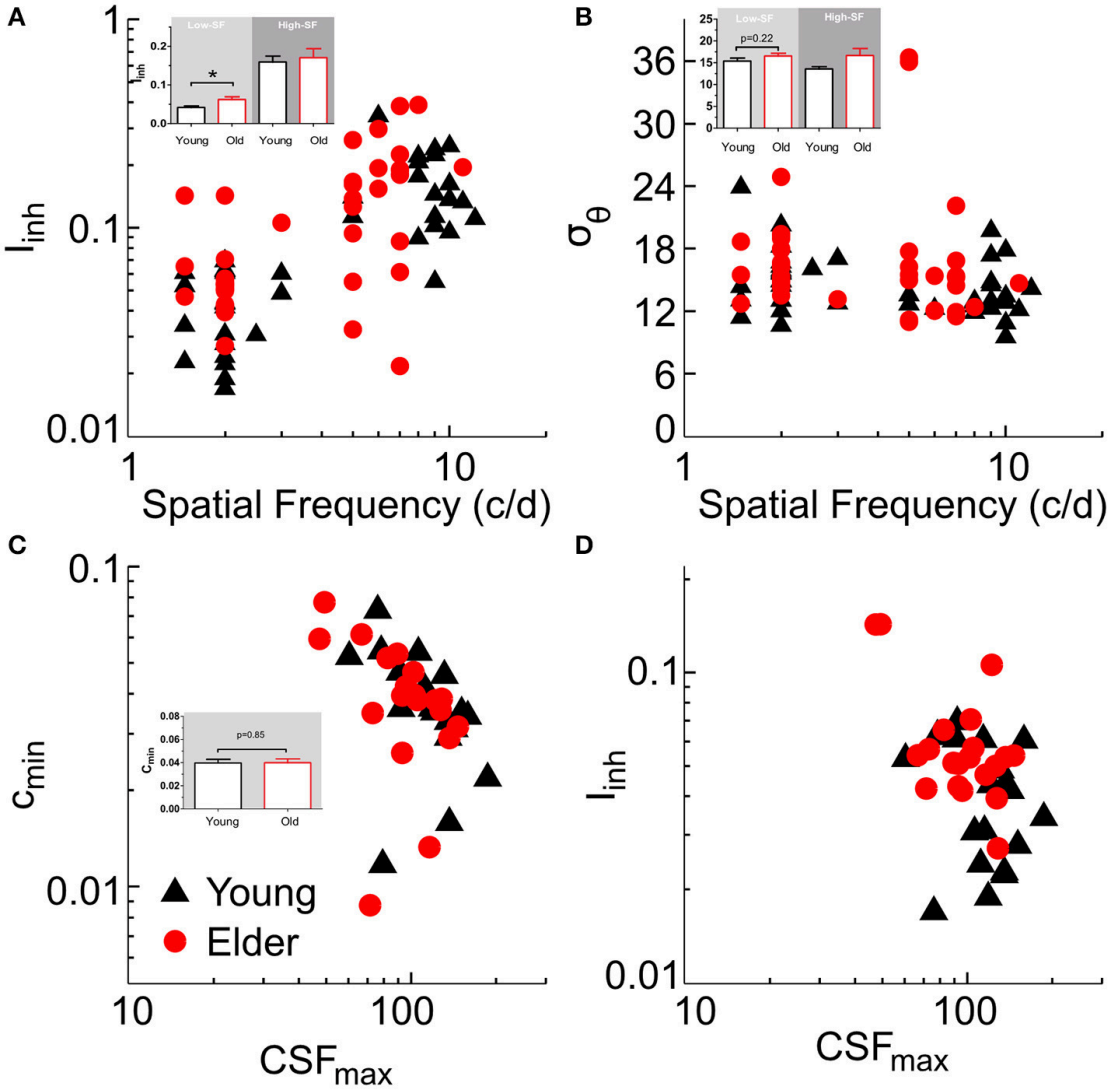
Model fitting results. Summary plots of subjects model parameters (I_inh_, σ_θ_, c_min_) obtained from tilt repulsion data **(A,B)** and contrast sensitivity data **(C)** and relations to the SF **(A,B)** or peak contrast sensitivity **(C,D)**. Insets depict histograms of the corresponding variable on the ordinate with significance values for the low-SF case.

We fitted the model first to tilt perception data (two free parameters: *I*_*inh*_, σ_θ_), and then to CSF data by using lateral inhibition as a fixed parameter (for CSF prediction, there were three free parameters: *c*_*min*_*, a, b*; with *c*_*min*_ corresponding to the best neuronal contrast sensitivity across all SFs; e.g., Figure 4E, peak value; see Methods for fitting details). The model was fit to the tilt repulsion data of ±15 and ±30° that provided independent estimates of orientation tuning widths and surround to center inhibitory amplitudes at both low- and high-SFs for each subject (Figures 5A,B). Age differences in tilt repulsion near the peak of perceptual sensitivity (low-SF) were explained by stronger lateral inhibition in the aged group [*t*_(38)_ = 2.95, *P* = 0.0054, log-transformed values], while no difference in orientation tuning widths were observed [*t*_(38)_ = 1.25, *P* = 0.22]. Additionally, both parameters co-varied with the SF at which it was measured across all population: lateral inhibition increased with higher SFs (*r* = 0.65, *P* = 4.4^*^10^−11^, *n* = 80), while neuronal tuning widths tended to decrease with higher SFs (*r* = −0.31, *P* = 0.0053, *n* = 80). This last finding resonates with the established physiological findings of sharper orientation tuning widths for cells with higher preferred SFs (Tolhurst and Thompson, 1981; De Valois et al., 1982), and provides strong support for our modeling approach. The model fit to the CSF data provided the minimum semi-saturation constant of the contrast response function near the peak of the CSF. Age group did not show differences in this optimal neuronal contrast sensitivity [*t*_(38)_ = −0.19, *P* = 0.85, log-transformed values; Figure 5C], but instead strongly correlated with the peak contrast sensitivity of the subjects (young group: *r* = −0.67, *P* = 0.001; old group: *r* = −0.51, *P* = 0.022). On the other hand, lateral inhibition was not correlated with peak contrast sensitivity (young: *r* = −0.27, *P* = 0.24; old: *r* = −0.37, *P* = 0.11; Figure 5D).

This combined modeling of orientation identification and contrast detection shows that neuronal contrast sensitivity and lateral interactions are not straightforwardly associated to the perceptual outcome and need simultaneously to be considered. Aging affected the strength of lateral interactions while individual subjects differences in neuronal contrast sensitivities still reliably represented their individual perceptual sensitivities.

### Neurophysiological data re-analysis

The model fitting results showed that orientation tuning widths are not systematically changed with aging, as proposed by previous behavioral reports (Betts et al., 2007; Delahunt et al., 2008; Govenlock et al., 2009), which we sought could be confirmed by orientation response properties of V1 neurons recorded from younger and older animals. Our published neurophysiological evidences from monkeys and cats showed that orientation processing in V1 is substantially affected by aging according to changes in orientation and direction bias (OB and DB) calculated by vector summation (Leventhal et al., 2003; Hua et al., 2006; Fu et al., 2010, 2013). It is difficult to directly compare orientation tuning widths from model results and neurophysiological data from these previous published analyses because these later did not consider the orientation tuning width of neurons (see Supplementary Material-5). Consequently, we re-analyzed our neurophysiological data through curve fitting, which allowed to separate effects onto the parameters of tuning: tuning width, minimum firing rate, and amplitude. This provided a clearer picture about age-related changes in orientation tuning properties.

**Figure 6:**
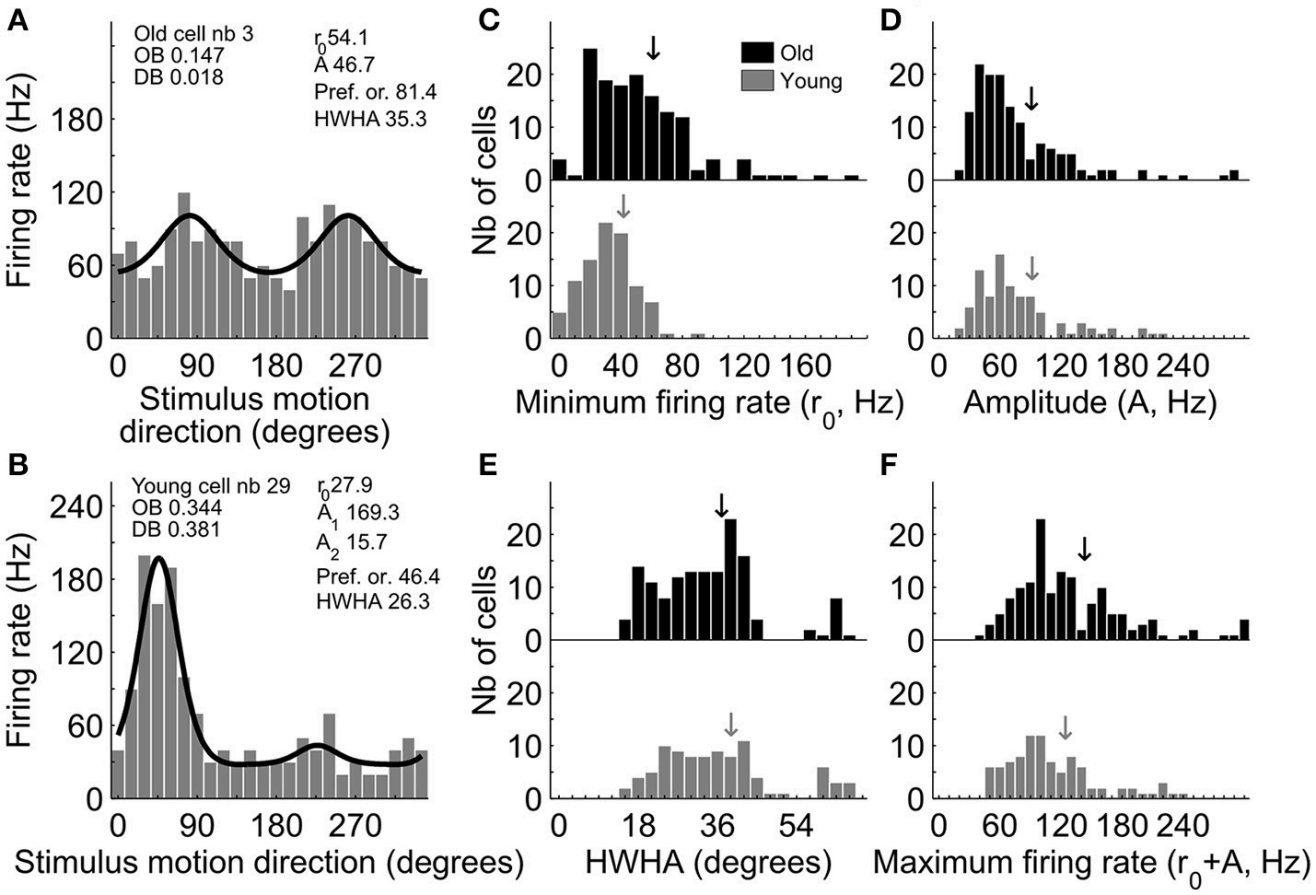
Neurophysiological data re-analysis of orientation tuning. Examples of fitting results for orientation **(A)** and direction **(B)** of motion selective cells; OB, orientation bias index, DB, direction bias index. Distribution of minimum firing rate **(C)**, amplitude **(D)**, *HWHA* **(E)**, and maximum firing rate **(F)** for the young and old cells. Arrows depict mean values.

Here, we re-analyzed one data set (Fu et al., 2013) by fitting direction/orientation tuning functions to each neuronal data set (Figures 6A,B). This data set contained 264 cells from 10 senescent monkeys and 140 cells from 4 young monkeys. We first tested whether a given neuron's data can be considered as tuned to orientation/direction of motion with an *F*-test (Mazurek et al., 2014; Tzvetanov, 2016). Then, we extracted three important parameters of the tuning curve from the tuned cells: the minimum firing rate in the tuning curve (*r*_0_), amplitude of firing (*A*), and tuning width as half-width at half-amplitude (*HWHA*).

Re-analysis results showed that in older monkeys, 143/264 (54.17%) neurons were found as tuned, a proportion which significantly differed (χ^2^ = 5.014, *P* < 0.05) from the 92/140 (65.71%) tuned cells in the younger animals. This confirmed the previous reports of smaller number of orientation tuned cells in V1 of senescent animals (Schmolesky et al., 2000; Leventhal et al., 2003; Hua et al., 2006; Fu et al., 2013). Importantly, *r*_0_, which here we consider as neural noise, was substantially increased [young: 32.83 ± 1.80, old: 52.11 ± 2.68; *t*_(226.51)_ = 5.28, *P* < 0.001], but amplitude *A* (young: 79.47 ± 4.60; old: 77.66 ± 4.29) and tuning width *HWHA* (young: 35.73 ± 1.37; old: 33.85 ± 1.00) were similar [*A*: *t*_(233)_ = −0.28, *P* = 0.78; *HWHA*: *t*_(233)_ = −1.13, *P* = 0.26] in the two age groups of neurons (Figures 6C–E). A control analysis on a subset of the data showed that these results were not due to possible confounding factors such as mixing different single units together (see Supplementary Material-4). Additionally, the maximum firing rate of the cells (*r*_0_+*A*) in the two populations (young: 112.30 ± 4.86; old: 129.77 ± 4.88) differed [*t*_(233)_ = 2.42, *P* < 0.05; Figure 6F], which was mainly attributed to the change in *r*_0_. The key results described above were also confirmed, out of the amplitude effect, by re-analyzing a second data set (Fu et al., 2010) from our laboratory about orientation/direction tuning and surround effects onto CRF (see Supplementary Material-3).

## Discussion

Neurophysiological studies on orientation processing in the primary visual cortex of animals have proposed that inhibitory function generally declines with age, and thus healthy older humans might have worse early perceptual abilities than younger persons. However, various psychophysical reports on aging have shown no changes in perceptual capabilities of orientation processing or even opposite effects, where interpretation leads to stronger inhibition. Firstly, we investigated these issues through a combination of psychophysical and modeling approaches. It was found that the amount of center-surround tilt repulsion, attributed to lateral inhibitory interactions and local orientation tuning widths, in the older group was higher compared to younger observers, and that contrast sensitivity of older adults was also globally lower than younger adults. We found a common explanation of all these phenomena in a single model of V1 that dissociated lateral and local inhibitory effects on the perceptual outcome of tilt perception. Our behavioral results and computational modeling provide important evidence that low-level processing deficits in the visual system of elders could be attributed to stronger lateral inhibition. Additionally, the modeling predicted that orientation tuning width in V1 globally should not change with aging. Therefore, secondly, we re-analyzed previous physiological data published by our laboratory and found that the neuronal data followed that prediction. That is, orientation tuning widths seemed globally stable across age, while the minimum firing rate of the tuning curve had a strong increase. Thus, our psychophysical modeling and re-analysis of neurophysiological results consistently revealed a detail picture of age-related changes in orientation processing, which we think solved some contradictions between neurophysiological and behavioral reports, and uncovered a differential age-related effects on local inhibition.

Lateral inhibition between neural mechanisms tuned to different orientations in V1 was proposed as an explanation of the repulsive TI already more than 40 years ago (Blakemor et al., 1970; Blakemore et al., 1973; Georgeson, 1973). An overall increase in illusory bias of tilt observed in the elderly group suggested an increase in lateral inhibition within V1 during aging of early visual function, which was confirmed by the model results. Our results are in line with recent reports that demonstrated an increase in perceptual surround suppression of contrast, orientation and other features in the older adults when compared to younger observers (Karas and McKendrick, 2015; Nguyen and McKendrick, 2016). We reproduced these previous contrast surround suppression results with our own stimuli by conducting a center-surround contrast discrimination task for all participants in our study, which correlated to the tilt biases of the subjects (see Supplementary Material-1). Thus, the similarity in results and interpretation of these two indexes of tilt repulsion and contrast suppression support the idea that they allow to measure a common neural mechanism, and we propose that both could be interpreted as stronger lateral inhibition. This stronger lateral inhibition of older adults should be dissociated from the standard interpretation of reduced local inhibition.

The model, based on the tilt repulsion data, also predicted that the orientation tuning widths of neurons (corresponding to local inhibition; Somers et al., 1995; Shapley et al., 2003) were similar between older adults and the younger population. These findings account for psychophysical results that sensitivities for orientation processing of older adults are not systematically and substantially changed when compared to younger adults (Betts et al., 2007; Delahunt et al., 2008; Govenlock et al., 2009). In one study (Govenlock et al., 2009), using a typical orientation masking paradigm that relates to the underlying orientation tuning, the authors did not find differences in tuning widths between groups of young and old persons, as in our results. Although our raw orientation thresholds were different between the two aged groups, these raw results should not be misinterpreted as evidence of differences, due to no adjustment of these values to the individual sensitivity. Indeed, the previous reports (Delahunt et al., 2008) showed that orientation discrimination thresholds are similar across age groups once they are adjusted for inter-subject variability of detection sensitivity, and thus age, which we could not perform due to lack of measurement in our experimental design. Instead, our methods allowed us to infer the underlying theoretical population tuning widths through the modeling of the tilt repulsion effect (Tzvetanov, 2012).

The computational prediction that orientation tuning width of neurons were unchanged in older adults when compared to younger populations was confirmed by re-analyzing our previous neurophysiological data collected in young and senescent macaques. These neurophysiological data was collected from classical receptive field measures, which relates to local inhibition in our model. The results showed an increased minimum firing rate of the tuning curve and spontaneous activities, which we consider here as neural noise, and maintained orientation tuning widths with aging. Additionally, the agreement of results with a second data set leads to a consistent and deeper understanding of neuronal tuning changes and aging effects onto orientation processing (see Supplementary Material-3). These outcomes suggest that the two levels of local inhibition, an untuned inhibition affecting neural noise and a tuned inhibition influencing orientation tuning width (Somers et al., 1995; Shapley et al., 2003), were changed by aging differentially, with a weaker untuned local inhibition but a maintained tuned inhibition. These findings revealed a more complex picture of age-related effects in the local inhibitory circuits than previously thought.

At the neurophysiological level, previous studies from different laboratories reported an age-related increase in spontaneous neuronal activity in macaque V1 (Leventhal et al., 2003; Wang et al., 2005; Zhang et al., 2008), cat A17 (Hua et al., 2006; Wang et al., 2014), rat A17 (Wang et al., 2006), and rat primary auditory area A1 (Hughes et al., 2010). The higher spontaneous activities in older animals cannot be accounted for with possible technical issue of differential effects of anesthesia upon cortical function in young and old animals. This possibility has been examined previously in both NHP (Schmolesky et al., 2000; Leventhal et al., 2003; Nguyen and McKendrick, 2016) and cats (Hua et al., 2006). The properties of individual cells, in both old and young animals, were recorded while systematically varying anesthesia and paralytic levels. It was found that giving as much as four times the minimum level of general anesthesia or paralytic required to anesthetize or paralyze both old and young animals does not alter the degree of selectivity for orientation and direction exhibited by V1 cells. Strong increase in anesthesia decreased neuronal responsiveness similarly in both groups. In addition, a greater sensitivity of old animal to anesthesia is hard to reconcile with the findings that old cells exhibit higher spontaneous firing than do young cells. Thus, differences of anesthesia effects in old animals does not seem of a strong concern. One mechanism hypothesized to underlie such age-related increase in spontaneous neuronal activity was a reduction of neuronal local inhibition due to diminished GABAergic neurotransmission (Leventhal et al., 2003; Wang et al., 2005; Zhang et al., 2008). Since weaker orientation tuned inhibition should strongly broaden the tuning widths (Somers et al., 1995; Shapley et al., 2003), which we did not observe, we propose that a strong weakening of untuned inhibition with aging provides an interpretation of the previously observed changes, while the tuned inhibition remains intact.

However, lateral inhibition, which involves long-range interactions among neurons, seems to increase with aging. The weak GABA mediated local inhibition might result in much higher background firing rates. Hence, a speculative possibility is that an increase in the lateral inhibition is created to counterbalance the decline in local inhibition. However, this “chain of effects” hypothesis needs further research.

In view of previous reports showing a decrease in surround suppression in older adults during motion discrimination task (Betts et al., 2005), the finding of older adults displaying a stronger center-surround inhibition in TI supports the proposal that there is an inhibitory process in the “static” domain of V1 that is different from the mechanism of dynamic motion integration in MT. Physiological studies on primate motion processing reported that center-surround receptive fields are particularly common in MT (Born and Bradley, 2005; Anton-Erxleben et al., 2009) except the input layer IV (Raiguel et al., 1995), which is one of the evidences indicating that surround inhibition observed in motion direction discrimination paradigm involved area MT and might not be inherited from its feed-forward inputs. Some findings (Tadin, 2015) also showed that there seem to be a direct relationship between area MT/V5 and spatial suppression in motion direction discrimination paradigm. Additionally, they found that subjects' performance in motion discrimination task did not change when V1 processing was disrupted through TMS pulses. On the other hand, lateral inhibition in V1 between neural mechanisms tuned to different orientations was suggested to underlie the repulsive TI (Blakemor et al., 1970; Georgeson, 1973). Thus, these two psychophysical measures seem to estimate different aspects of cortical function and seem to reflect independent neuronal mechanisms.

Last, it is noteworthy to mention that the current study on aging brings together different methods and results obtained from different species, humans and non-human primates. It would be an important step forward to be able to demonstrate in a single observer the link between the neurophysiological changes and their putative perceptual consequences. Unfortunately, there is no study about the behavioral changes on orientation discrimination abilities of senescent NHP subjects yet. We know of one recent study demonstrating visual decline in old NHP, but animals performed a simple fixation task with biologically irrelevant stimuli (Csete et al., 2015). At our best knowledge, such a work in elder studies is missing, we think because of the vulnerability of elder animals. Currently, we are only able to compare human behavioral data and physiological analyses from other species.

In conclusion, our behavioral, computational and physiological findings provide a new perspective on aging of the visual system. We unveiled two different types of aging-related changes at the primary visual cortex, increase in lateral inhibition and unchanged local orientation coding capacities, but associated to higher neuronal noise. This suggested that local and lateral inhibition were differently affected by aging, and explained disparate results among previous behavioral and neurophysiological studies.

## Materials and methods

### Subjects

The experiment included two groups: 21 younger adults (19 young adults of 20–30 years, plus co-author TT who was 41 years old) and 21 older adults (65–79 years). One young subject data was excluded due to no clear staircase convergence in the TI task (very high thresholds). One elder's data was excluded because of incorrect task performance in the replication of the contrast discrimination task (staircases without convergence properties). Subjects younger than 30 years old were students of the University of Science and Technology of China, while others were recruited from local communities. All subjects were naive to the purpose of the experiments (except the author), and their informed consents were obtained before participation. Examination (MMSE) was performed on older adults to exclude probable dementia. Alcoholism, stroke and depression were also exclusion criteria by questionnaires before conducting experiments. Participants also provided information about their general health, to exclude people with systemic conditions known to affect visual function (for example, diabetes, migraine, schizophrenia, and epilepsy) or who were taking medications known to affect visual function (e.g., anti-anxiety or anti-depressant medications). All participants were measured with normal or corrected-to-normal visual acuity ]younger = 0.016 ± 0.050, elder = 0.013 ± 0.071 (logMAR); mean ± std]. This research has been approved by the ethics committee of University of Science and Technology of China and followed the guidelines of the Declaration of Helsinki. Subjects were paid for their participation hourly.

### Set-up

The experiment was conducted in a dimly illuminated room. Stimuli were displayed on a 40.0 × 30.0 cm CRT monitor (Sony G520; 85 Hz, resolution of 1,600 × 1,200 pixels) with self-programmed Matlab functions (Mathworks Inc.) using the Psychophysics toolbox (Brainard, 1997). Stimuli were displayed using an NVIDIA Quadro K600 graphics system and viewed binocularly. To avoid local cues for vertical/horizontal and position, the screen was delimited by a 30.0 cm diameter circular window cut in a black cardboard (Tzvetanov, 2012). Luminance values were obtained with the help of the contrast box switcher (Li et al., 2003), that allowed to extend luminance range digitization above 10 bits, and thus provided the necessary minimum contrast step for CSF measure. Calibration was performed each day.

The eye-to-screen distance was maintained with a chin rest and fixed at 4 meters for CSF measure and the experiments for TI test with high-SF, and 2 m for TI test at low-SF. Experiments were initiated by subjects with a keyboard press. Subjects were requested to fixate on a small black square on the screen center and stimuli would be presented 200 ms after fixation point disappearance. Subjects responded by pressing corresponding keyboard keys.

### Stimuli

Stimuli used for CSF measure were vertical sine-wave gratings with different SFs (0.71, 1, 1.41, 2, 2.83, 4, 5.66, 8, 11.31, 16, and 22.63 cycle/degree) (Figure 1A). All stimuli subtended 2° aperture, and were presented on the center of monitor with 40 cd/m^2^ background. A border-mask blended the stimuli to the background to minimize edge effects. There were 275 trials in total (25 trials/SF). Each trial consisted of two 100 ms intervals that separated by a 500 ms inter-stimulus interval (ISI), and each interval was announced by a prior short beep. The target sine-wave grating was presented in one of the two intervals.

Stimuli used in the tilt illusion (TI) experiments were a central Gabor patch (test grating) surrounded by an annulus of sine-wave grating (inducer) with different surround orientation (SO: 0°, ±15°, ±30°, ±75°; angle was defined with respect to the orientation of the center test) (Figure 1B). The orientation of the central Gabor patch was varied around vertical from trial to trial to measure subject's perceived verticality. The Gabor patch was defined through the following equation:

(1) $$\begin{array}{l} L\left(x,\;y\right)=\;Lo+LoC\;\exp\;(-(x^{2}+y^{2})/\sigma^{2})\\ \;\times \cos(2\pi f(xcos\theta +ysin\theta )) \end{array}$$

with *L*_0_ the background luminance of the screen, *C* the Gabor patch contrast, and *f* and θ its spatial frequency and angle relative to vertical. The size of the stimuli were scaled with SF, keeping the central test stimulus fixed at 4 cycles. Surround annulus width was equal to center test diameter. Both test and inducer had same spatial frequency (SF) and constant contrast (90%) and were presented on a mean background luminance of 35 cd/m^2^. Specifically, TI was measured under two SF values, a low-SF located around peak contrast sensitivity, a high-SF which smaller than cutoff SF but higher than low-SF. Each participant had his/her own low-SF and high-SF values, obtained from CSF curve individually. Low-SF and high-SF conditions were conducted in two separate blocks and each block consisted of 420 trials (60 trials/SO). The stimulus in each trial was presented for nine video frames (~100 ms) and no feedback was provided.

### Procedure

Before formal measure, each subject received a short practice session (about 80 trials) for each task. A CSF measure was conducted prior to TI measure to extract low-SF and high-SF values. TI measure for high- and low-SF were conducted sequentially in two separate blocks.

A 3-key response design (Kaernbach, 2001) was used for CSF measure, with two keys for responding in which interval the subjects perceived the signal and a third key for undecided decision. The contrast of the target sine-wave grating was varied in multiple of 10% according to the up-down staircase procedures, with correct responses followed by decreasing contrast with 4.5/1.5 steps and incorrect responses by increasing contrast of 8/7 steps for SFs of 2n (with n from ½ to 4½ in steps of 1, and from 1 to 4 in steps of one respectively). Undecided key presses were randomly drawn as correct/incorrect. Starting points were contrasts of 0.5,192 0.005, 0.5, 0.005, 0.5, 0.005, 0.5, 0.005, 0.7 0.05, 0.8 for the successive 11 SFs, respectively, and each staircase “down” step-size was additionally 3 times bigger for the first 4 trials (example results in Figure 1C). Feedback was provided by different sound for correct and incorrect responses and mute for the undecided key.

The 3-key design was also used for TI measure where the observer was required to report whether the orientation of the center test was clockwise (CW) or counter clockwise(CCW) from his/her internal vertical standard by pressing two predefined keys. The third key was allowed if the subject felt he/she did not perceive central target grating (due to surround suppression; especially at high SFs), and “not seen” cases were randomly drawn as CW or CCW for the staircase procedure. The center orientation was varied according to the weighted up-down rule with steps up/down of 5/2 and 2/5 degrees with base step of 1°. Staircases started at the opposite side of the convergence point allowing rapid measures within the transition region of the psychometric function. No feedback for any responses.

### Data analysis

We used Bayesian fitting to adjust theoretical psychometric functions to the CSF and TI data (Treutwein and Strasburger, 1999).

#### Contrast sensitivity function

A 2D psychometric function was fit to the 2D contrast-SF (*c, f*) data, with the probability of correct response defined as:

(2) $$\begin{array}{l} P\left(c,\;f\right)\text{​}=\text{​}\gamma \text{​}+\text{​ }\frac{1-\gamma -\lambda }{1\text{​}+\text{​}\exp(-\log\left(\frac{21}{4}\right)\text{​}(\log\left(c\right)-\log\left(\frac{1}{S\left(f\right)}\right))/\sigma } \end{array}$$

with parameters γ and λ being subject's “guess rate” (see below) and lapse rate, and 2σ defining the spread between 16 and 84% of the function in the range γ to 1-λ (assuming constant spread, σ, across SFs). *S(f)* is the standard 3-parameters sensitivity function (Rohaly and Owsley, 1993):

(3) $$\begin{array}{l} S\left(f\right)=Mf^{a}\exp\left(-\frac{f}{b}\right) \end{array}$$

used in previous studies to define the CSF shape in the SF dimension. The 3-key design data was processed following Garcia-Perez (García-Pérez, 2010), which in the event the subject followed the 3rd key instructions allows a decrease in measurement variability. The Likelihood function is then:

(4) $$\begin{array}{l} \text{log}ML=\left(\sum_{i}y_{i}\log(p(c_{i},f_{i})\right)+\left(1-y_{i}\right)\log(1-p(c_{i},f_{i})))\\ \;+\left(\sum_{k}1/2\log(p(c_{k},f_{k})\right)+1/2\log(1-p(c_{k},f_{k}))) \end{array}$$

with the first sum (trials with index *i*) running over all responses 1st or 2nd interval and the second sum (trials with index k) running over the “undecided” 3rd key responses. The variables in the above equation are: (*c*_*i*_*, k, f*_*i*_,_*k*_)–contrast & SF pair presented at trial *i* or *k, y*_*i*_–correct/incorrect (1/0) response of subject at contrast & SF levels (*c*_*i*_*, f*_*i*_), *p* (*c*_*i*_*, k, f*_*i*_, _k_)-theoretical probability of correct response (psychometric function). In this ML equation, the first term is the standard log-ML term for fitting binomial data; the second “Fechner” term is simply log (*p(1-p)*), the logarithm of the binomial variability at the stimulus levels for which the subject pressed “undecided”/“not seen”; it is maximized when *p*(*c*_*k*_*, f*_*k*_) is 0.5, i.e., when the subject is totally uncertain about the interval of signal presentation, and thus provides a firm theoretical ground for introducing “undecided”/“not seen” responses into the 2AFC design. The lapse rate was fixed at 1% for all but one subject where it was zero. The “guess rate” γ was 0.5. An example CSF fit for one subject and his responses to each presented stimulus is displayed in Figure 1C.

*Tilt repulsion*. We fit a 1D psychometric function to the orientation discrimination data for each surround orientation, with probability of CW responses to target orientation θ given by:

(5) $$\begin{array}{l} P\left(\theta \right)=\lambda +\frac{1-2\lambda }{1+\exp(-\log\left(\frac{21}{4}\right)(\theta -a)/\sigma } \end{array}$$

where λ is subject's lapse rate, and *a* and σ being the perceived vertical orientation (also called “bias”) for the given surround and the threshold of the subject for perceiving a deviation from verticality, respectively. Because of the symmetry in the experimental design (symmetric surrounds of ±15, ±30, and ±75°), for the fitting we imposed that thresholds of opposite surround orientations (e.g., −30 and +30°) are the same. The lapse rate was fixed at 0% for low-SF condition, and 1% for high-SF. The data was processed by eliminating any datum with 3rd key responses (subject did not see the target), and we computed the amount of surround suppression as the proportion of 3rd key presses. Bias values were computed as the half-difference between two opposite surround orientations.

### Statistical analysis

For contrast sensitivity, a two factors between-within ANOVA (age group × spatial frequency) was conducted. For TI, a 3-way between-within subject ANOVA (age group × surround orientation × spatial frequency) was conducted on all data. For center-surround contrast discrimination task, a *t*-test was used to analyze the suppression ratio data (paired or unpaired, as appropriate). All statistical levels use Geisser-Greenhouse epsilon-hat adjusted values, where appropriate. Spearman rank correlation was used. Data were expressed as mean ± SE.

### Model

The model was developed for another study in our laboratory, concerning Amblyopia deficits (Huang et al., 2017), and a detailed description was given in the accompanying manuscript (Huang et al., 2017). We provide the model description as in the original work for consistent methodological description in the current work.

### Simple model of V1 surround-to-centre interactions

We assume, as in many previous studies, that simple feature perception as local orientation and contrast can be explained through the decoding of primary visual cortex neuronal activities. Therefore, we investigated a simple V1 model of two-layer neurons coding the main features of interest in the study: orientation, contrast, SF, and space. The model consists of orientation hyper-columns arranged into a hexagonal structure, with each hyper-column containing neurons responding to various contrasts and SFs. First layer neurons can be thought of simple cells whose responses are as follows:

(6) $$\begin{array}{l} r_{ijk}\left(\theta ,f,c\right)=A\times T\left(\theta ;\theta_{i}\right)\times F\left(f;f_{j}\right)\times C\left(c;c_{k}\right) \end{array}$$

with “preferred” features (θ_*i*_,*f*_*j*_,*c*_*k*_) and the three normalized tuning functions to orientation, SF and contrast are described as wrapped-Gaussian (Swindale, 1998), log-Gaussian (Yuan et al., 2014), and hyperbolic ratio (Albrecht and Hamilton, 1982), respectively (*A* is the maximum amplitude of firing of the neuron). They are:

(7) $$\begin{array}{l} T\left(\theta ;\theta_{i}\right)=e^{-\frac{1}{2}{\left(\frac{\theta -\theta_{i}}{\sigma_{\theta }}\right)}^{2}} \end{array}$$

(8) $$\begin{array}{l} F\left(f;f_{j}\right)=e^{-\frac{1}{2}{\left(\frac{f-f_{j}}{\sigma_{SF}}\right)}^{2}} \end{array}$$

(9) $$\begin{array}{l} C\left(c;c_{k}\right)=\frac{c^{n}}{c_{k}^{n}+c^{n}} \end{array}$$

Remark: for the contrast tuning, *c*_*k*_ is the semi-saturation constant and can be called the “preferred” contrast of the neuron, since for contrasts around *c*_*k*_ the neuron is the most informative above the input contrast (Chirimuuta and Tolhurst, 2005; May and Solomon, 2015a,b), and away from *c*_*k*_ it asymptotes and provides no information about contrast input.

These simple cells feed the second layer of neurons through a spatial (excitatory center)–(inhibitory surround) connectivity structure, whose responses *R*_*ijk*_ follow the conductance-based model (Grossberg, 1988; Piëch et al., 2013):

(10) $$\begin{array}{l} R_{ijk}\left(\theta ,f,c\right)=h\left(v_{ijk}\right) \end{array}$$

(11) $$\begin{array}{l} \tau \frac{dv_{ijk}}{dt}=-v_{ijk}+\left(v_{e}-v_{ijk}\right)g_{e}+\left(v_{i}-v_{ijk}\right)g_{i} \end{array}$$

(12) $$\begin{array}{l} g_{e}=\underset{mno}{\sum }\omega_{ijk,mno}^{cen}r_{mno} \end{array}$$

(13) $$\begin{array}{l} g_{i}=\underset{mno,\left(x,y\right)}{\sum }\omega_{ijk,mno,\left(x,y\right)}^{sur}r_{mno,\left(x,y\right)} \end{array}$$

with *h()* a transducer (rectifying) function transforming voltage to firing rate and feature weights ω's defined as:

(14) $$\begin{array}{l} h\left(v\right)=m\times \max\left(0,v-T\right) \end{array}$$

(15) $$\begin{array}{l} \omega_{ijk,mno}^{cen}=I_{c}G_{im}^{c}G_{jn}^{c} \end{array}$$

(16) $$\begin{array}{l} \omega_{ijk,mno,\left(x,y\right)}^{sur}=I_{s}G_{im}^{s}G_{jn}^{s}G_{x,y} \end{array}$$

and the various parameters are: *T* is the voltage threshold of firing, *m* is the slope of voltage-to-firing rate relation, τ is the cell time constant, *v*_*e*_ and *v*_*i*_ are the excitatory and inhibitory equilibrium voltage potentials, *g*_*e*_ and *g*_*i*_ are respectively the excitatory and inhibitory conductances feeding the corresponding neuron through a weighted sum of first layer activities (*g*_*e*_ sum is within hyper-column; *g*_*i*_ sum is over all surrounding hyper-columns), *G*_*im, jn*_ are Gaussian tuned feature weights [respectively within orientation and within SF; with possible different tuning widths indexed (*c,s)*], *G*_*x, y*_ is a spatial weight function summing surrounding hyper-columns activity, and *I*_*c, s*_ are the center/surround excitatory/inhibitory input strengths, respectively. Here, it is assumed that the weights are independent across features and iso-feature tuned (peaking at the receiving neuron preferred value (*i*,*j*), i.e., iso-orientation and iso-SF).

In the feed-forward model equation (11) can be analytically solved, giving:

(17) $$\begin{array}{l} v_{ijk}=\frac{v_{e}g_{e}+v_{i}g_{i}}{1+g_{e}+g_{i}} \end{array}$$

(18) $$\begin{array}{l} R_{ijk}=h\left(v_{ijk}\right) \end{array}$$

Using all relations above and an input with uniform surround (all surrounding hyper-columns are stimulated with the same stimulus of orientation θ_*s*_ and contrast *c*_*s*_), assuming the center stimulus (θ_*c*_,*c*_*c*_) excites the center hyper-column, the center input excitatory/inhibitory conductances can be analytically computed:

(19) $$\begin{array}{l} g_{e,i}=\frac{I_{c,s}A_{c,s}}{\sigma_{\theta }^{c,s}\sigma_{SF}^{c,s}}\overset{c,s}{\underset{\theta }{\sum }}\;B_{C,S}\times e^{-\frac{1}{2}\frac{{\left(\theta_{i}-\theta_{c,s}\right)}^{2}}{{\left(\sigma_{\theta }^{c,s}\right)}^{2}+\sigma_{\theta }^{2}}}\times e^{-\frac{1}{2}\frac{{\left(f-f_{j}\right)}^{2}}{{\left(\sigma_{SF}^{c,s}\right)}^{2}+\sigma_{SF}^{2}}} \end{array}$$

The various constants are defined as:

(20) BC,S=((∑SFc,s)2(log(2))22+Kc,slog(2))∑SFc,se

(21) $$\begin{array}{l} {\left(\overset{c,s}{\underset{\theta }{\sum }}\right)}^{-2}={\left(\sigma_{\theta }^{c,s}\right)}^{-2}+{\left(\sigma_{\theta }\right)}^{-2} \end{array}$$

(22) $$\begin{array}{l} {\left(\overset{c,s}{\underset{SF}{\sum }}\right)}^{-2}={\left(\sigma_{SF}^{c,s}\right)}^{-2}+{\left(\sigma_{SF}\right)}^{-2} \end{array}$$

(23) $$\begin{array}{l} K_{c,s}\left(f;f_{j}\right)=\frac{{\left(\sigma_{SF}^{c,s}\right)}^{2}\log\;f+\sigma_{SF}^{2}\log\;f_{j}}{{\left(\sigma_{SF}^{c,s}\right)}^{2}+\sigma_{SF}^{2}} \end{array}$$

where $\sigma_{\theta }^{c,s}$ and $\sigma_{SF}^{c,s}$ are the orientation and SF tuning widths of the weight functions *G*_*im*_ and *G*_*jn*_ for center-center and surround-to-center connections, respectively, *A*_*c, s*_ are the contrast-weighted amplitude of firing of the input neurons *r*_*ijk*_ for center/surround respectively, *I*_*c, s*_ are the excitatory and inhibitory weight amplitudes, and

(24) $$\begin{array}{l} I_{s}=n_{s}\times I_{inh} \end{array}$$

defines the total inhibitory input from all surrounding hyper-columns with mean inhibitory strength per hyper-column *I*_*inh*_, respectively; *n*_*s*_ is a “mean” number of surround hyper-columns influencing the center.

### Fixed parameters

Summary of the set of parameters that were fixed in the model (following a “normalized” conductance based subtractive inhibition model, e.g., Piëch et al., 2013): *n*_*s*_ = 6, *n* = 2, *A* = 2, σ_*SF*_ = 1, *m* = 1, *T* = 1, *I*_*c*_ = 1, *v*_*e*_ = 14/3, *v*_*I*_ = −2/3. SF dimension sampling was every ¼ octaves from ½ up to 64 c/d, orientation feature sampling every 2°. The contrast tuning relation was kept normalized by multiplying its amplitude by a factor *(1* + *c*${}_{k}^{n}$*)*.

### Model based fitting of CSF and tilt perception data

Here we investigate how based on the output activity *R*_*ijk*_ of the network we can predict the perception of the subject in the two main features of interest, contrast detection for predicting the contrast sensitivity function and orientation identification for predicting the tilt repulsion effect. It is assumed that perception is based on decoding of the center hyper-column activities as described below.

#### Modeling the contrast sensitivity function (CSF in 2D)

In this experiment, the target stimulus is a vertical and uniform sine-wave grating limited in a circular spatial window, whose strength (contrast) is varied in order to measure the perception threshold across all SFs.

Given the uniform input stimulus, all neurons *r*_*ijk*_ have exactly the same input and thus their activation across orientation, SF, and contrast have the same profiles and peaks (for any *i*,*j*,*k:* $r_{ijk}^{c}=r_{ijk}^{s}$). Second, given the task of detecting always a vertically oriented grating, by assuming that subjects disregard other orientated activities through an unspecified attentional mechanism, we simplified the term over orientations in equation (19) into a value of one (in practice, this simplification can be thought of pooling these orientation neuronal activities into the constants *I*_*c, s*_ or *A*_*c, s*_) and modeled only one orientation of network activities.

In the above model description, one important function in predicting the contrast sensitivity at a given SF is the hyperbolic ratio of the neuronal population (Chirimuuta and Tolhurst, 2005; May and Solomon, 2015a,b). We assume that contrast detection across all SFs is performed by decoding the activities of the neurons with the smallest semi-saturation constant $c_{k}^{\min}\left(SF\right)=\min\left(c_{k}|SF\right)$. In our model, to predict the CSF across SFs we additionally need to properly describe the relation between $c_{k}^{\min}$ and SF together with SF tuning width versus preferred SF. Based on previous neurophysiological reports (see Supplementary Material-2), we fixed:

(25) $$\begin{array}{l} c_{k}^{\min}\left(f_{j}\right)=c_{\min}\times \frac{{\left(ab\right)}^{a}e^{-a}}{f_{j}^{a}e^{-\frac{f_{j}}{b}}} \end{array}$$

(26) $$\begin{array}{l} \sigma_{SF}=e^{-\frac{f_{j}-2}{0.2}} \end{array}$$

where *f*_*j*_ is the preferred SF of the neuron and parameters (*c*_*min*_,*a*,*b*) define the neuronal sensitivity function across SFs. We fixed $\sigma_{SF}^{c,s}=\sigma_{SF}/2$ because of center/surround tuning widths entanglement in predicting behavioral results, *I*_*c*_ = 1; *n*_*s*_ = 6, and neurons with *c*_*k*_ > 1,000 were pruned.

Last step, for predicting the contrast sensitivity function across SFs, we used the standard signal detection theory, defining the psychometric function:

(27) $$\begin{array}{l} \text{P}\left(c,f\right)=\gamma +\left(1-\gamma -\lambda \right)P_{th} \end{array}$$

(28) $$\begin{array}{l} P_{th}=2\left(\int_{0}^{+\infty }\frac{R_{j}-R_{0}}{\sqrt{Var\left(R_{j}\right)+Var\left(R_{0}\right)}}dR_{j}-0.5\right) \end{array}$$

with the lapse and guess rates obtained as described in the above section Data analyses, and *R*_0_ being the activity of the neurons for no signal input.

From the above model description, there are only four free model parameters that need to be adjusted for predicting the full CSF: *c*_*min*_, *I*_*inh*_, *a, b*. It was done by replacing equation (2) with equation (27) and following all remaining steps.

#### Modeling orientation identification (tilt illusion)

For this feature, we make a different set of simplifications in the model feature space based on the experimental design for tilt perception. Here, center and surround hyper-columns are stimulated with varying orientations while the contrast of the center and surround stimuli are kept constant and equal. Therefore, we can describe the two-layer neuronal network activities through the above mathematical development. But here, we fixed the input layer contrast activation at *A* = 2 and *c* = 1 (near maximum contrast, e.g., Piëch et al., 2013), and given the task of identifying the orientation of the center stimulus for a fixed SF (Georgeson, 1973), we simplified into equation (19) the term over SFs by assuming subjects disregard other SF neuronal activities (eg through an unspecified attentional mechanism), giving:

(29) $$\begin{array}{l} g_{e,i}=\frac{I_{c,s}A_{c,s}}{\sigma_{\theta }^{c,s}}\overset{c,s}{\underset{\theta }{\sum }}\left(e^{-\frac{1}{2}\frac{{\left(\theta_{i}-\theta_{c,s}\right)}^{2}}{{\left(\sigma_{\theta }^{c,s}\right)}^{2}+\sigma_{\theta }^{2}}}\right) \end{array}$$

with all constants defined in model description section. We fixed the center and surround feed-forward orientation tuning widths to $\sigma_{\theta }^{c,s}=\sigma_{\theta \;}/2$.

Last, to predict the orientation psychometric function (tilt data), we decoded the perceived orientation of the stimulus [*a* in Equation (5)] as the vector average orientation of the center hyper-column activities (Gilbert and Wiesel, 1990; Seung and Sompolinsky, 1993; Tzvetanov, 2012). For this feature, there are only two free parameters, *I*_*inh*_ and σ_θ_, that are sufficient to provide tilt misperception description (Gilbert and Wiesel, 1990; Tzvetanov, 2012). Lapse rate was fixed at 1%.

### General fitting procedure

First, each individual data set was fit with the tilt prediction part only for SO of ±15 and ±30° in order to extract *I*_*inh*_ at both high and low SFs. For a given SF, a single discrimination threshold for all SOs was set as free parameter. Then, the parameter *I*_*inh*_ was used as fixed in the CSF fitting procedure in order to find the best three parameters (*c*_*min*_, *a, b*) that described subject's CSF data.

### Neurophysiological data re-analysis

The direction of motion tuning curves were fit with a wrapped von-Mises function of the form:

(30) $$\begin{array}{l} r\left(\Delta \theta_{1}\right)=b+a_{1}\exp\left(k\left(\cos\left(2\Delta \theta_{1}\right)\right)-1\right)\\ r\left(\Delta \theta_{2}\right)=b+\left(a_{1}-a_{2}\right)\exp\left(-2k\right)\\ \;+a_{2}\exp\left(k\left(\cos\left(2\Delta \theta_{2}\right)-1\right)\right) \end{array}$$

where r is the response of the neuron, and the parameters *b, a1, a2*, and *k* allow to compute the physiological parameters of interest, minimum firing rate, the two amplitudes to opposite direction of motions, and half-width at half-amplitude, as:

(31) $$\begin{array}{l} \;r_{0}=b+a_{1}\exp\left(-2k\right)\\ \;A\left(\theta_{pref}\right)=a_{1}\left(1-\exp\left(-2k\right)\right)\\ A\left(\theta_{pref}+180\right)=a_{2}\left(1-\exp\left(-2k\right)\right)\\ \;HWHA=0.5\cos^{-1}(1+\log\frac{1+\exp\left(-2k\right)}{2})/k) \end{array}$$

The two variables Δ_θ_ are defined as Δθ_1_ = θ*-*θ_*pref*_ and Δθ_2_ = θ*-*θ_*pref*_-*180*, each defined in the range ±90°, and the additional term for *r*(Δθ_2_) allows continuity at the boundary. The fitting was done by minimizing the χ^2^ between the data and the model function, and because standard errors were not present, it was assumed *se*_*i*_ = (FANO × *y*_i_/5)^½^ with FANO = 1.5, and for the few cases where *y*_*i*_ = 0, *se*_*i*_ = 0.5477. We performed three successive fits: (1) an orientation tuned function fixing *a*_1_ = *a*_2_ (4 free parameters: *b, a, k*, θ_*pref*_); (2) a direction tuned function leaving parameters *a*_1_ and *a*_2_ independent; and (3) a flat-topped version of the equation (Albrecht and Hamilton, 1982) allowing for more peaked or flatter types of tuning curves, that included one more parameter ν [in equation (30), Δθ_1,2_ is replaced by Δθ_1,2_/2 + ν sin(Δθ_1,2_)]. Multiple refits were done with random starting points for finding the best set of parameters. Each fitted curve was then used to test, with a nested *F*-test (Tzvetanov, 2016), whether it described the data better than the global mean of the data, i.e., whether the cell could be described as tuned. Then, if two or more functions described the data better than the mean, we further used a nested *F*-test to test whether the more complex functions (with additional parameters) described the data better than the simpler one, and selected the corresponding model. In the models with different amplitudes to opposite motion direction, the amplitude of firing was defined as the higher of the two. In the flat-top model, the half width of half amplitude (HWHA) was directly searched in a discretized direction space (0.1° steps). At the end, we additionally eliminated any fitted curve with too narrow of tuning (*HWHA* < 15), which discarded 8 old cells and 7 young cells. Two example fits are presented on Figures 6A,B for one orientation and one direction tuned cells, together with their orientation and direction bias values. Here, we were not interested of the overall goodness-of-fit of the model to the data. Nevertheless, in Supplementary Material-4, we show that there are no essential differences in fit quality between old and young cell types once variability is properly accounted for.

### Neurophysiological experimental materials and methods

Please see Fu et al. (2013) for detailed information regarding electrophysiogical experiments in monkeys. Briefly:

#### Animals

Subjects for this study were 14 male rhesus monkeys (Macaca mulatta) classified into 2 groups: the young (YA) group consisted of 4 monkeys who were 5.8 ± 0.5 (mean ± standard deviation) years old; the old group included early senescent (ES) group (7 monkeys at ages of 21.6 ± 1.5 years) and late senescent (LS) group (3 monkeys at ages of 29.3 ± 2.3 years). Monkeys were examined ophthalmoscopically and had no indication of optical or retinal problems that would impair visual function. Retinal blood vessels, lens clarity, and the maculae were all within the normal range.

#### Animal preparation and recording

Subjects were sedated with Ketamine HCl (10–15 mg/kg, i.m., ParkeDavis, Morris Plains, NJ, USA) and then anesthetized with 3–5% halothane (Halocarton Laboratories, River Edge, NJ, USA) in a 70:30 mixture of N_2_O: O_2_. Intravenous and tracheal cannulae were inserted. Animals were then placed in a stereotaxic frame. All pressure points and incisions were infiltrated with a long-acting local anesthetic (2% lidocaine HCl; Copley Pharmaceuticals, Canton, MA, USA). A craniotomy was performed above the area in V1 corresponding to the representation of central vision. After surgical procedures were finished and sufficient level of anesthesia was assured, a mixture of d-tubocurarine (0.4 mg/kg/h; Sigma, St. Louis, MO, USA) and gallamine triethiodide (7 mg/kg/h; Sigma) was infused intravenously to induce and maintain paralysis. During recording, animals were artificially ventilated. End tidal CO_2_ partial pressure was monitored and maintained at ~4–4.5%. Body temperature was maintained at 38–38.5°C by an automatically regulated heating pad. The anesthesia was maintained with a mixture of N_2_O (70%), O_2_ (30%), and halothane (0.25–1.0%, as needed). Electrocardiography was continuously monitored. The level of anesthesia was assessed by periodically applying nociceptive stimulation (toe pinch) and monitoring the changes in the heart rate.

The nictitating membrane was prepared with neosynephrine. The pupils were dilated with atropine, and the eyes were covered with contact lenses for protection from desiccation. The optics of the eyes and retinal vasculature were monitored throughout the experiment. No visible deterioration in optics occurred during the experimental period in any of the animals. Action potentials of isolated units were recorded extracellularly by glass microelectrode (filled with 2-M N_a_Cl, 1–3-MΩ impedance at 1000 Hz), which was advanced by a hydraulic microdrive (David Kopf Instruments, Tojunga, CA, USA).

#### Visual stimulation

For each isolated single unit, the eye preference was determined and all subsequent stimuli were presented monocularly to the dominant eye. Cell's receptive field was carefully plotted on a tangent screen by hand with the use of an ophthalmoscope. After that, a 17″ CRT color monitor (85-Hz refresh rate; Sony, Tokyo, Japan) was positioned at the distance of 57 cm in front of the animal's eyes and centered on the receptive field of the cell. The program to generate the visual stimuli was written in MATLAB (MathWorks, Natick, MA, USA), using the extensions provided by the high-level Psychophysics Toolbox (Brainard, 1997) and low-level Video Toolbox (Pelli, 1997). To quantify the orientation and direction selectivity of V1 cell, drifting bars were used, whose width, length, and moving speed were adjusted to elicit strongest response from the recorded cell. The direction of motion of each presented bar was orthogonal to its orientation. We used moving bars at 24 randomly chosen movement directions, ranging from 0° to 360° in steps of 15° to compile the tuning curves for the cells studied. Each direction was presented 10 times. The inter-trial interval was 2–5 s. The luminance of the stimuli used was 39 cd/m^2^ for white and 0.95 cd/m^2^ for black.

#### Data collection

Signals recorded from the microelectrode were amplified (1,000×), band-pass filtered (300–10,000 Hz), and then digitized (sampling frequency of 20 kHz) by using an acquisition board (National Instruments, Austin, TX, USA) controlled by IGOR software (WaveMetrics, Portland, OR, USA). Such original signals were stored in a computer for offline analysis. The responses to moving bars were defined as the maximal value in the peristimulus time histogram (PSTH, bin width of 10 ms) during the stimulation period. Before each drifting bar was presented, the spontaneous (baseline) activity was recorded during a period of 0.5–0.7 s while the screen with average luminance was presented.

## Ethics statement

This study was carried out in accordance with the recommendations of the ethics committee of University of Science and Technology of China with written informed consent from all subjects. All subjects gave written informed consent in accordance with the Declaration of Helsinki. The protocol was approved by the the ethics committee of University of Science and Technology of China.

## Authors contributors

ZW and TT designed and analyzed behavioral experiments; ZW performed experiments; ZW and TT performed data analysis and neurophysiological re-analysis; SY and YF provided physiological data and discussed its re-analysis; TT performed modeling; ZW, YZ, and TT provided project supervision and funds; ZW and TT wrote the paper; all authors discussed and commented on the manuscript.

### Conflict of interest statement

The authors declare that the research was conducted in the absence of any commercial or financial relationships that could be construed as a potential conflict of interest.
