# Supplementary material for: Aging Potentiates Lateral but Not Local Inhibition of Orientation Processing in Primary Visual Cortex
Source: Front Aging Neurosci. 2018 Feb 5;10:14. doi: 10.3389/fnagi.2018.00014 (PMC5807380; doi:10.3389/fnagi.2018.00014)
Supplement: Supplementary file 1 [file Presentation1.PDF]

Supplementary Material

Aging Potentiates lateral but not local inhibition of orientation processing in primary visual cortex

Zhengchun Wang, Shan Yu, Yu Fu, Tzvetomir Tzvetanov*, Yifeng Zhou*

*Correspondence:

Tzvetomir Tzvetanov (tzvetan@ustc.edu.cn) and Yifeng Zhou (zhouy@ustc.edu.cn)

Table of Contents

1. Correlation between tilt illusion bias and contrast suppression ratios 1
   Methods 1
   Results 2
2. Neurophysiological accounts of V1 neurons tuning relations 3
3. Re-analysis of the data set of Fu et al. (2010) 4
4. Control tests of the main text results 6
5. Relating previous publications results with the current analysis 7
References 8

1. Correlation between tilt illusion bias and contrast suppression ratios

Methods

Subjects and set-up are same to the CSF and tilt illusion measures. The eye-to-screen distance was 2 m for the replication study of contrast discrimination.

The stimuli used in center-surround contrast discrimination experiments consisted of a small central Gabor patch of vertical orientation (f=4 c/deg, equ.4 in main text), with its diameter fixed at 4 cycles. It was presented either alone or surrounded by an annulus of sine grating (4 c/deg, inner radius equals center Gabor patch radius, outer radius is inner radius plus center diameter). All phases were fixed at zero. Stimuli were presented on a mean background luminance of 35 cd/m2. The stimulus with center alone was a test Gabor patch whose contrast was varied in order to measure the perceived contrast of the center when it is flanked by the surround. The center-surround stimulus had a predefined set of possible contrasts. The full measurements included all combinations of contrasts for center-surround among the three values of 20%, 40% and 80%. This was performed in order to compare our design to a recent study (Karas & McKendrick, 2015). For the present report we restrict our presentation to the center-surround of 80%-80% that matches closely the tilt illusion condition.

The procedure for contrast discrimination task was as follows: one block consisted of 300 trials measuring the three psychometric functions for the center-surround stimulus with three reference center contrasts and surround contrast fixed within the block. In each trial, there were two intervals, separated by 500 ms, presenting in one interval the central grating alone and in the other the central grating surrounded by an annulus. The stimuli were randomly assigned to intervals. Each stimulus was accompanied by a short beep and presented for 100 ms. No feedback was provided. The test contrast was varied with the weighted up-down staircase procedure (Kaernbach, 1991) and for each reference contrast four staircases were ran with steps up/down of 7/1.5, 1.5/7, 6/2.5, and 2.5/6 of the baseline step of 5% contrast change. Starting points were 0.05, 0.9, 0.05 and 0.9 respectively. The 2AuFC task with three key responses was used where the observer was required to nominate in which interval

(first/second) the central patch had the highest contrast. The third key was used by observers if they could not identify in which interval the grating had higher contrast and "indecision" cases were randomly drawn as the test perceived lower/higher contrast.

For data analysis of center-surround contrast perception, the 80%-80% condition was fit with a logistic psychometric function:

$$p(c) = \gamma + \frac{1 - \gamma - \lambda}{1 + \exp(-\log(21/4)(\log(c) - \log(a))/\sigma)} \quad \text{(Equ. 1)}$$

representing the probability to respond test Gabor patch (without surround) had higher contrast. We used the "Fechner's" definition for undecided key presses into the maximum-likelihood equation ("undecided" cases were considered as half one category and half the other, e.g. Garcia-Perez & Alcala-Quintana, 2011):

$$\text{logML} = \left(\sum_i y_i \log(p(c_i)) + (1 - y_i)\log(1 - p(c_i))\right) + \left(\sum_k \frac{1}{2}\log(p(c_k)) + \frac{1}{2}\log(1 - p(c_k))\right), \quad \text{(Equ. 2)}$$

with the second sum indexed *k* running over the "undecided" key presses. The psychometric parameters γ and λ were constrained as follows: γ was constrained between 0 and 0.5, while λ was constrained between 0 and λ_{max} (with λ_{max}=1-γ-(1-2γ)/2/(1+exp(log(21/4)log(a)/σ)) in order to have $p(c=1)\geq 0.5$), and both had flat prior. The second constrain was necessary because we found that multiple subjects (16/40) had difficulties to discriminate the two central patches for test targets (no surround) of contrasts around 90% (near the maximum available), and disambiguate parameters entanglement (that is, their psychometric functions were not saturating near c=1). A suppression ratio was calculated (perceived contrast/physical reference contrast) to quantify the strength of the centre-surround interactions(Karas & McKendrick, 2015). A value below 1 indicates surround suppression, while a value above 1 indicates enhancement.

Results

A recent report demonstrated stronger surround suppression in the elder when compared to young population (Karas & McKendrick, 2015). Using our stimuli, a center-surround contrast discrimination task was conducted for all participants in order to investigate how it correlated to our own findings with the tilt repulsion. In our results, the suppression ratios were lower than 1 for both groups (Supp.Fig.1), and the elder's ratios were significantly lower than the younger adults ($t(38)=4.48$, $p<0.0001$), thus replicating their finding (Fig.7 in Karas & McKendrick, 2015). If the two psychophysical measures probe the same underlying visual system, we should expect a correlation between them. To analyse the relationship between tilt bias and contrast suppression ratios, the expected orientation bias at 4 c/d (Bias(SF=4)) for each individual subject was extracted from a line regression between orientation bias and log-SFs (Fig.3a,b in main text). There was a significant negative correlation in the younger group at both surround orientations (Suppl.Fig.1A: $r=-0.77$, $p<0.0001$ at 15°; $r=-0.64$, $p<0.001$ at 30°) and in the elder group at 15°(Suppl.Fig.1B: $r=-0.51$, $p<0.05$ at 15°; $r=-0.25$, $p=0.29$ at 30°).

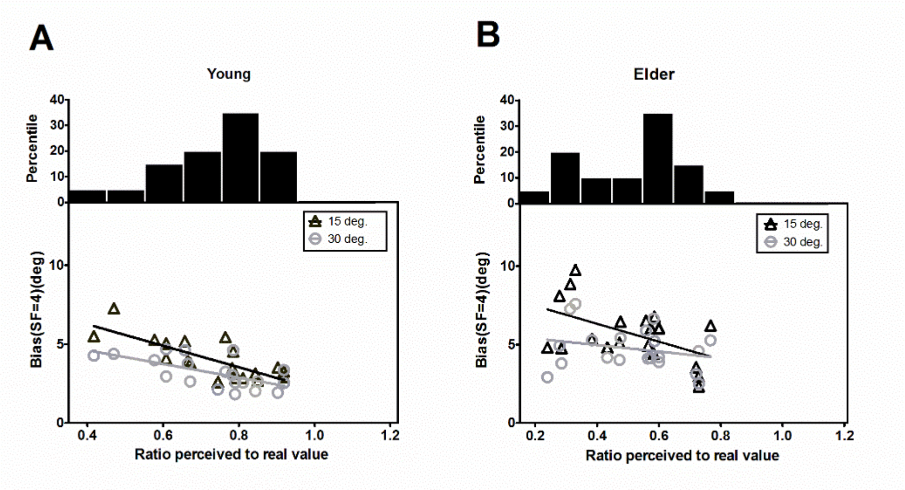

Supplementary Figure 1 | Correlation between tilt repulsion & contrast suppression ratio. (A and B) Correlation between tilt repulsion (bias, at spatial frequency equals 4 c/d and contrast surround suppression ratio (ratio of perceived contrast/physical reference contrast) for younger (A) and elder adults (B).

2. Neurophysiological accounts of V1 neurons tuning relations

The simple model of V1 cells we tested in the study is based on multiple tuning characteristics of each cell: orientation, SF and contrast. These tuning functions are characterized very well among different animal species, and it was found that the parameters between or within these tunings co-vary. Since our model uses these properties, we had to fix the relations between the characteristic parameters, which we did based on the various physiological reports. Here, we summarize the literature knowledge used for model parameters relations.

About the contrast tuning, the CRF was chosen as the usual hyperbolic ratio function (Albrecht and Hamilton, 1982; see also Methods in main text, equ.10):

$$r(c) = \frac{c^n}{c^n + c_k^n}. \qquad \text{(Equ. 3)}$$

In this equation, the most important variable for our model is the semi-saturation constant c_k, normally representing the contrast at which the cell reaches half of its maximum amplitude (but see end of this part). One issue with this variable is that one has to define how c_k changes when the power n changes. These two variables were found to covary across the neuronal population (see re-analysis statement of D.Ringach's monkey data in the Methods of Chirimuuta & Tolhurst, 2005; or Persi et al., 2011, for an in-depth analysis and consequences of these relations). Since we need the relation only between the neurons with lowest c_k (best sensitivity at a given SF), we fixed n=2 for all neurons. Second, neuronal sensitivity was found to vary with the preferred spatial frequency of the neuron (Albrecht and Hamilton, 1982). Further, for the purpose of CSF modelling, it is known that the envelope of the population of neuronal sensitivities varies with the SF in a similar way as the CSF theoretical equation (Equ.6 and Equ.17 in Methods of main text). Supplementary Fig. 2 plots data from two studies providing monocular neuronal sensitivities vs. preferred SF (cat: Anzai et al., 1995; amblyopic monkeys: Kiorpes et al., 1998). The bell-shaped upper boundary of the data is well described by the standard CSF equation, and thus it was used to describe the neuronal sensitivity function in the model. Data were extracted from Kiorpes et al. (1998) (amblyopic monkeys LF, FP and OC) and Anzai et al. (1995; cat data). The envelope of the best sensitive

cells shows the typical ogive shape also present in CSF (example fit over upper data points across SFs in panel LF).

Last point that we would like to emphasize, is that the standard hyperbolic equation used in the literature has the disadvantage that for low powers of n or increasing c_k, the semi-saturation constant does not represent the contrast at half-maximum any more. Thus, one has to recompute the half-amplitude constant, c½, as:

$$c_{1/2}^n = c_k^n/(1 + 2c_k^n) \quad \text{(Equ. 4)}$$

This parameter is also displayed in the main text Fig. 4e,g with the arrows.

We fixed the SF tuning width σ_{SF} to decrease with higher preferred SFs f_j (Equ.18 in Methods of main text), as reported physiologically by various authors (De Valois et al., 1982; Kulikowski and Bishop, 1981; Tolhurst and Thompson, 1981).

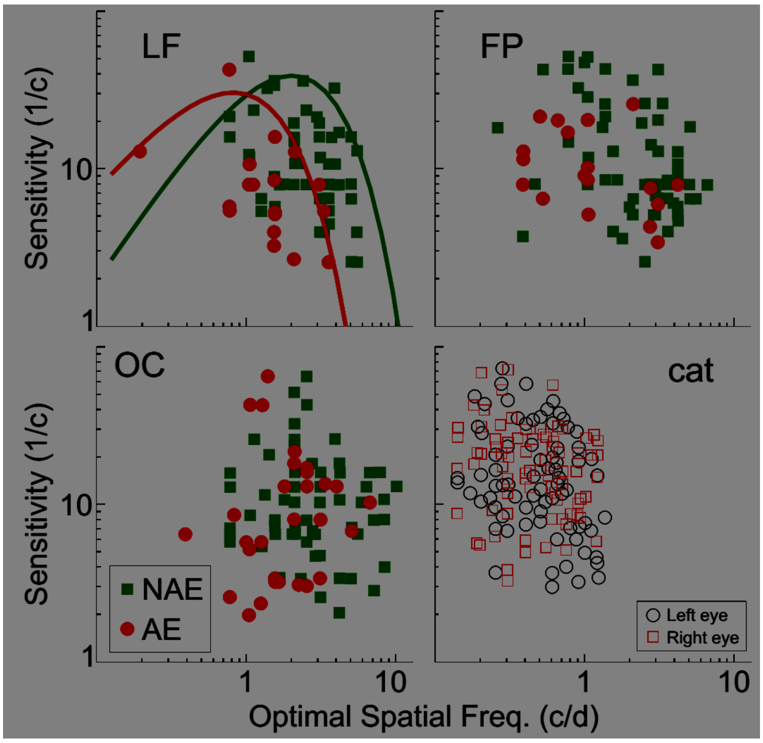

Supplementary Figure 2 | Contrast sensitivity versus optimal/preferred spatial frequency of the cells extracted from the corresponding studies (Kiorpes et al., 1998 - LF, FP, OC; Anzai et al. - cat). The study of Kiorpes et al. (1998) provides three anisometropic monkeys neuronal sensitivity data (NAE/AE – non amblyopic eye/amblyopic eye).

3. Re-analysis of the data set of Fu et al. (2010)

In their article, Fu et al. analysed possible changes with aging of surround suppression onto the orientation tuning properties of the classical receptive field (CRF) (e.g. Gilbert & Wiesel, 1990) measured on young and old monkeys (4 young monkeys mean age 5.5 years and 3 old monkeys with mean age 28 years; for further methodological details please refer to the article). They performed it by presenting luminance sine gratings into the CRF first for characterizing the standard orientation/direction tuning, and then in one second condition,

they presented the optimal center orientation in the surround and re-measured the orientation tuning of the cell. The results were interpreted and analyzed with respect to the old-fashioned orientation bias/direction bias (OB/DB) indexes, which introduce unwanted interpretation pitfalls (Mazurek et al., 2014; Tzvetanov, 2016).

Here, as done in the main text for the data set obtained with bar stimuli, we first re-analysed the main CRF orientation/direction tuning parameters. They are presented in Supplementary Figure 3. In the old cells, 20 out of 46 cells were found tuned while in the young cell population 66 out of 81 were tuned; this difference was highly significant (Z=4.4, $p<0.0001$). The minimum firing rate r_0 in the old cell population (61.3±4.7) was significantly higher than the young cells (30.6±2.6)(Wilcoxon rank sum test: Z=4.8, $p<0.00001$); the amplitudes A in the old cells (37.1±4.1) were found significantly lower than those of the young cells (63.7±4.4)(Z=-3.08, p=0.0021); *HWHA* were not different between old (44.5±3.2) and young cells (41.2±1.6)(Z=1.21, p=0.23); and the total amplitude of firing (r_0+A) was similar between old (98.4±6.5) and young (94.3±0.6) cells (Z=1.10, p=0.27). Overall, this data set confirms the main text finding of minimum firing rate change and no tuning width change, while the amplitudes seem to be differentially modulated by the type of stimulus used for the measures.

This data set was also interesting because the authors directly investigated in old and young cells the tuning with the presence of optimal surround orientation, known to inhibit the activity of the center. We also fitted this condition with the orientation/direction tuning functions. In this condition, we discovered a puzzling behavior of the cells orientation/direction tuning, especially in the old cells. From the 46 old cells only 14 cells were found tuned in this condition, and from those, only 6 cells were simultaneously tuned in the standard CRF tuning measure (previous paragraph). That is, we found that 8 cells "popped-out" as being tuned with the presence of the surround while they were not in the standard measure (no surround). For the young cells, 35/81 were orientation tuned in the surround condition, and among them only 2 cells were not tuned in the standard condition. These results are partly consistent with the original article conclusion based on the OB/DB indexes (orientation bias/direction bias) that there are even smaller number of orientation/direction tuned cells when surround is present, but also with the main finding in the previous paragraph about the tuning parameters r_0 and *HWHA* (means±s.e. for surround condition; r_0: young – 36.2±3.1, old – 64.8±6.4; *HWHA*: young – 37.1±2.1, old – 42.0±4.1), but not for the amplitudes A (young: 42.7±3.9; old: 35.8±4.8).

We would like to emphasize that the apparent inconsistency of the original interpretation of "decreased strength of surround suppression" in the cells of senescent animals with the re-analysis presented here is due to the old-fashioned way of analysis through the OB/DB indexes or other composite variables (Mazurek et al., 2014; Tzvetanov, 2016), which are obtained from a combination of tuning parameters. On the contrary, one has to use a clear statistical test for presence of tuning for each individual cell and then analyze the tuned cells parameters by discarding from the analysis the cells that are not tuned. In the current analysis, from the statistical test and tuning parameters changes, the exact nature of stronger/weaker suppression in the older cells seems to us difficult to ascertain for multiple reasons. First, in both old and young cells, the number of tuned cells when the surround was present was decreased, leading to the possibility of stronger surround suppression in both populations, which destroys orientation/direction tuning of the cell. Second, the mean amplitude of firing of the tuned cells with surround present seemed, when compared to the CRF tuning, unchanged in the old cells but lower in the young cells, which leads to the possibility that old cells might have "weaker" surround suppression than the young cells. Third, the mean minimum firing rates did not change between the two conditions, also leading to an interpretation of similar effects of surround in both conditions and cell types. Last, and importantly, the tuning widths in the surround-present condition for old and young cells were

globally similar to the no-surround condition, also supporting similar surround effects in both types of neurons.

The above outcome rose interesting questions which, sadly, the low number of cells in the data set and the puzzling behavior in the old cells preclude from any reasonable interpretation. Therefore, we did not go further in presenting results of this condition in detailed form and inferring plausible interpretations when surround is present.

To conclude this second data set re-analysis, we found that the minimum firing rate and tuning widths followed the results presented in the main text, that old cells exhibited higher minimum firing rates but no significant differences in tuning widths when compared to young cells. On the contrary, the amplitudes seem to be differentially affected as a function of the type of stimulus.

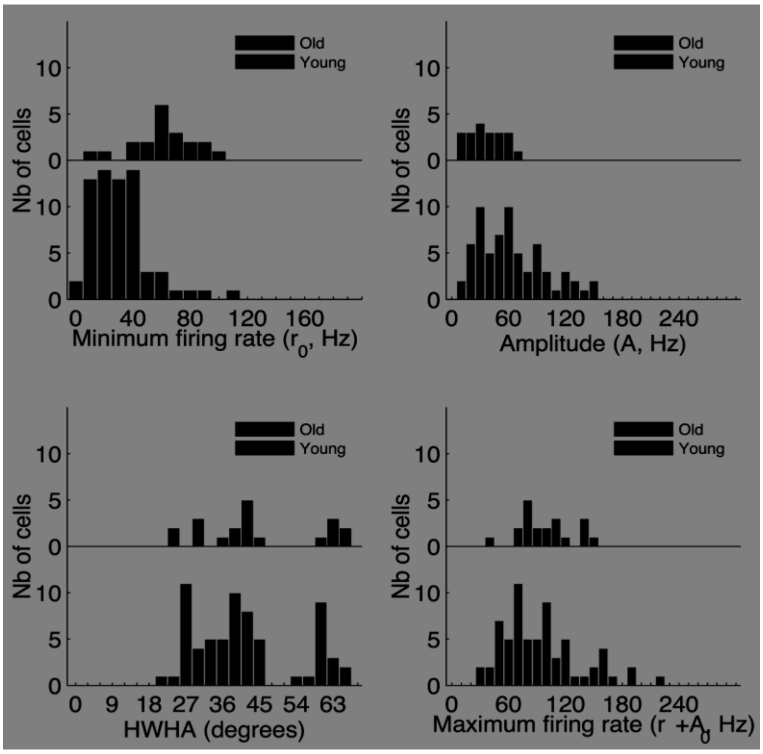

Supplementary Figure 3 | Results for the re-analysis of Fu et al. (2010) data set, measured with luminance grating stimuli in the classical receptive field. Format as Fig.6 in main text.

4. Control tests of the main text results

The main text results were obtained from all original data in Fu et al. (2012). Since this data set was not analyzed for possible presence of multi-unit activity (MUA), we considered that the tuning curves might be contaminated by multiple closely tuned cells. While we would expect this effect to be similar between the old and young cells because of the same methodology employed within the study and across monkeys, we decided to check for plausible contamination by restricting the main analysis to a subset of the cells with the best tuning widths. The idea is that these cells should be much less contaminated since they have the narrowest tuning widths. For that purpose, we selected the 30% cells with smallest tuning widths in each population (for old, n=43; for young, n=28) and re-applied the main text

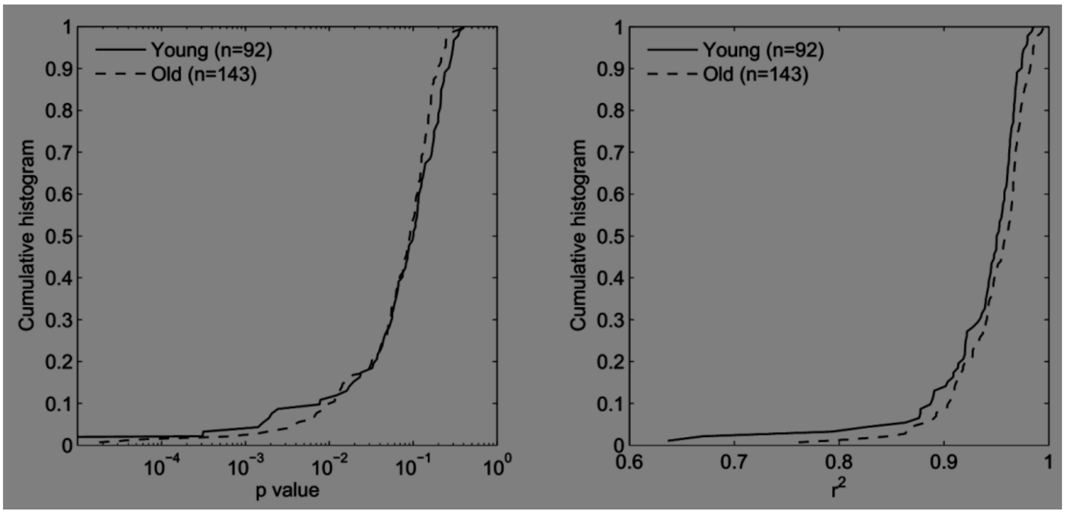

Supplementary Figure 4 | Goodness-of-fit test for the tuned cells in both type of neurons. Left panel: cumulative histogram for old and young cells of the probability to obtain each individual cell tuning data given its best fitted model. Right: cumulative histograms for old and young cells of the classical r^2 coefficient of determination.

comparisons on the three tuning properties. The minimum firing rate r_0 (young: 36.0±3.9; old: 54.1±5.7) was significantly different between the two populations (t(67.59)=2.61, p=0.011), while the amplitude A (young: 100.9±9.9; old: 89.7±9.6) and *HWHA* (young: 22.3±0.7; old: 20.9±0.6) were both not significantly different between the two cell types (A: t(65.0)=-0.81, p=0.42; *HWHA*: t(59.7)=-1.61, p=0.11). This restricted sub-sample analysis confirms the main text results, but on those cells that should not be considered as strongly affected by MUA.

Second, we asked whether the fit quality could be different between the two categories of cells, that is, we checked whether the tuned old cells might have worse tuning properties than the young cells through a goodness-of-fit measure. For that purpose, we first show the classical coefficient of determination, r^2, in both populations (Supplementary Figure 4, right panel). It demonstrates that the old cells have globally higher r^2 (Wilcoxon rank sum test for differences in medians: p=0.026), which is due to the aging effects onto the tuning characteristics. The increased minimum firing rate (r_0) provides globally higher firing rates for the old cells thus artificially inflating the total sum of squares and creating the impression of better model description of that data set. Therefore we sought a more appropriate way by computing for each cell the likelihood to obtain the given data set from the best fitted function based on the Poisson statistics for each datum, and re-transformed it into individual cell p-value by taking the likelihood to the power of (1/24). The cumulative histograms of these p-values are depicted on the left panel of Supplementary Figure 4. Once the variability is properly accounted for, the young and old cells did not show significant differences in goodness-of-fit (Wilcoxon test: p=0.31).

5. Relating previous publications results with the current analysis

Here, we briefly describe how the results reported in the previous publications from the laboratory should be related to the current analysis through orientation/direction tuning functions.

The previous reports used the orientation bias (OB) and direction bias (DB) indexes as a measure for the cells about presence of tuning to “orientation/direction”. The major outcome of the previous studies was that aging decreased the mean OB/DB of the cell population, thus showing a smaller number of orientation/direction tuned cells in the elder animals. Across the population of cells, this interpretation should be globally valid.

It is important to note that these indexes are difficult to interpret with respect to changes in

tuning properties (background, amplitude, or tuning width, see Mazurek et al., 2014; Tzvetanov, 2016), because of two important points. Firstly, the analysis based on them includes all neurons sampled during the study, even the ones that have a circularly flat response (i.e. they respond uniformly to all directions of the stimulus). Thus, a decrease/increase in OB/DB could reflect the fact that there are less/more tuned cells in the first condition when compared to the second, simply because the OB/DB of these not tuned cells is close to zero (but not zero). Secondly, even for the cells that have clear orientation/direction tuning, the decrease/increase of OB/DB does not provide information about what changed in the tuning property of the neurons. As a simple example, consider a theoretical Gaussian orientation tuned cell with minimum firing rate (r_0) of 10 Hz, amplitude (A) of 40 Hz, and σ of 25 degrees (giving *HWHA* of about 29.4 degrees). Its OB value, when sampled every 1 degree, is 0.40. If a second cell has simply a higher minimum firing rate of 30 Hz, and all other parameters and sampling are identical, it provides a value of 0.22 for its associated OB (but the other parameters also influence the final OB/DB estimate, see Mazurek et al., 2014; Tzvetanov, 2016). This demonstrates that a change of OB/DB alone is not informative about the nature of the underlying modification, whether there are less cells coding for orientation/direction or the parameters of the tuned cells changed.

The results of our previous publications are globally consistent with the idea that there are less tuned cells in the elder animals. But they could also be interpreted with the globally increased minimum firing rate of the old cells when compared to the young cells. These two effects are present in the data and thus influence the difference between population of young and old cells.

The previous publication results are consistent with our current analysis and interpretation, but here we go further and provide a deeper understanding about the exact nature of the changes that happen during aging. Additionally, our main behavioral and computational study made us mainly interested into one of the tuning parameters, the tuning width, that relates directly to the modeling outcome. Therefore, the physiological data reanalyses, while interesting *per se*, are not detailed further in the current work.

References

Albrecht, D. G. & Hamilton, D. B. Striate cortex of monkey and cat: contrast response function. Journal of Neurophysiology (1982), 48, 217-236

Anzai, A.; Bearse, M. A.; Freeman, R. D. & Cai, D. Contrast coding by cells in the cat's striate cortex: monocular vs. binocular detection. Visual Neuroscience (1995), 12, 77-93

Chirimuuta M, Tolhurst DJ. Does a Bayesian model of V1 contrast coding offer a neurophysiological account of human contrast discrimination? Vision research (2005), 45, 2943-2959.

DeValois, R. L.; Albrecht, D. G. & Thorell, L. G. Spatial frequency selectivity of cells in macaque visual cortex. Vision Research (1982), 22, 545-559

Fu, Y.; Wang, X.S.; Wang, Y.C.; Zhang, J.; Liang, Z.; Zhou, Y.F. & Ma Y.Y. The effects of aging on the strength of surround suppression of receptive field of V1 cells in monkeys. Neuroscience (2010), 169, 874-881

Fu, Y.; Yu, S.; Ma, Y.; Wang, Y. & Zhou, Y. Functional Degradation of the Primary Visual Cortex During Early Senescence in Rhesus Monkeys. Cerebral Cortex (2012) 23, 2923-2931

Garcia-Perez MA, Alcala-Quintana R. Interval bias in 2AFC detection tasks: sorting out the artifacts. Attention, perception & psychophysics (2011), 73, 2332-2352.

Gilbert, C. D. & Wiesel, T. N. The influence of contextual stimuli on the orientation

selectivity of cells in primary visual cortex of the cat. Vision Research, 1990, 30, 1689-1701

Karas, R. & McKendrick, A. M. Contrast and stimulus duration dependence of perceptual surround suppression in older adults. Vision Research (2015), 110, 7-14.

Kaernbach, C. Simple adaptive testing with the weighted up-down method. Perception & Psychophysics (1991), 49, 227-229

Kiorpes, L.; Kiper, D. C.; O'Keefe, L. P.; Cavanaugh, J. R. & Movshon, J. A. Neuronal correlates of amblyopia in the visual cortex of macaque monkeys with experimental strabismus and anisometropia. Journal of Neuroscience (1998), 18, 6411-6424

Kulikowski, J. J. & Bishop, P. O. Linear analysis of the responses of simple cells in the cat visual cortex. Experimental Brain Research (1981), 44, 386-400

Mazurek, M.; Kager, M. & van Hooser, S. D. Robust quantification of orientation selectivity and direction selectivity. Frontiers in Neural Circuits (2014) 8:92

Persi, E., Hansel, D., Nowak, L., Barone, P., and van Vreeswijk, C. Power-Law Input-Output Transfer Functions Explain the Contrast-Response and Tuning Properties of Neurons in Visual Cortex. Plos Computational Biology (2011), 7.

Tolhurst, D. J. & Thompson, I. D. On the variety of spatial frequency selectivities shown by neurons in area 17 of the cat. Proceedings of the Royal Society of London B (1981), 213, 183-199

Tzvetanov, T. Commentary: Robust quantification of orientation selectivity and direction selectivity. Frontiers in Neural Circuits (2016), 10
